# Myocyte enhancer factor‐2 and p300 interact to regulate the expression of homeostatic regulator Pumilio in *Drosophila*

**DOI:** 10.1111/ejn.14357

**Published:** 2019-02-21

**Authors:** Wei‐Hsiang Lin, Richard A. Baines

**Affiliations:** ^1^ Division of Neuroscience and Experimental Psychology School of Biological Sciences Faculty of Biology, Medicine and Health University of Manchester Manchester Academic Health Science Centre Manchester UK

**Keywords:** activity, homeostasis, neuron, promoter, transcription

## Abstract

Pumilio (Pum), an RNA‐binding protein, is a key component of neuron firing‐rate homeostasis that likely maintains stability of neural circuit activity in all animals, from flies to mammals. While Pum is ubiquitously expressed, we understand little about how synaptic excitation regulates its expression in the CNS. Here, we characterized the *Drosophila dpum* promoter and identified multiple myocyte enhancer factor‐2 (Mef2)‐binding elements. We cloned 12 *dmef2* splice variants and used a luciferase‐based assay to monitor *dpum* promoter activity. While all 12 dMef2 splice variants enhance *dpum* promoter activity, exon 10‐containing variants induce greater transactivation. Previous work shows dPum expression increases with synaptic excitation. However, we observe no change in *dmef2* transcript in larval CNS, of both sexes, exposed to the proconvulsant picrotoxin. The lack of activity dependence is indicative of additional regulation. We identified p300 as a potential candidate. We show that by binding to dMef2, p300 represses *dpum* transactivation. Significantly, *p300* transcript is downregulated by enhanced synaptic excitation (picrotoxin) which, in turn, increases transcription of *dpum* through derepression of dMef2. These results advance our understanding of *dpum* by showing the activity‐dependent expression is regulated by an interaction between p300 and dMef2.

**Abbreviations**
AD: activation domain
BD: DNA binding domain
CNS: central nervous system
dsRNA: double‐stranded RNA
FF: Firefly‐luciferase
Luc: luciferase
Mef2: myocyte enhancer factor‐2
Ren: renilla‐luciferase
PTZ: pentylenetetrazole
PTX: picrotoxin
Pum: Pumilio
PRE: Pum Response Element
SD: synthetic dropout
UAS: upstream activating sequence
UTR: untranslated region

## 1 INTRODUCTION

Pumilio (Pum), a founding member of the Pum/FBF (Puf) RNA‐binding protein family, is central to multiple aspects of CNS function, including (but not limited to) firing‐rate homeostasis, dendritic morphogenesis, synaptic growth and function, expression of acetylcholinesterase and long‐term memory (Chen et al., 2008; Driscoll, Muraro, He, & Baines, 2013; Menon et al., 2004; Muraro et al., 2008; Vessey et al., 2010). Despite a wide‐ranging involvement in many aspects of CNS function, little is understood concerning the regulation of Pum expression in the CNS. Importantly, reduced levels of Pum have been linked to seizure and epilepsy in *Drosophila*, rodents and human (Follwaczny et al., 2017; Lin, Giachello, & Baines, 2017; Siemen, Colas, Heller, Brustle, & Pera, 2011; Wu et al., 2015).

Pumilio binds an eight nucleotide sequence in mRNA (UGUANAUA, where N = A, G, C or U), termed a Pum Response Element (PRE) and, by doing so, induces translational repression (Arvola, Weidmann, Tanaka Hall, & Goldstrohm, 2017; Wharton, Sonoda, Lee, Patterson, & Murata, 1998; Wreden, Verrotti, Schisa, Lieberfarb, & Strickland, 1997). Pum‐dependent translational repression requires a number of coregulators, including Nanos (Nos) and brain tumour (Brat), which bind different, but equally characterized, RNA motifs to form a complex with Pum (Arvola et al., 2017). An analysis of 3'UTRs in the *Drosophila* genome identified 2477 transcripts containing one or more PREs highlighting the possibility that many transcripts undergo Pum‐mediated translational regulation. The number of transcripts regulated may, however, be considerably less because specificity is also likely provided by both PRE copy‐number and proximity of PRE‐, Nos‐ and Brat‐binding motifs within individual transcripts (Arvola et al., 2017).

The number of transcripts expressing PREs underscores the importance of Pum. Despite this, however, our understanding of *pum* expression and role(s) is limited and, where information is known, is mostly focused on post‐transcriptional modification. For example, the *dpum* transcript is itself regulated through translational repression by the cytoplasmic RNA‐binding Fox protein (Rbfox1, aka A2BP1) in order to promote germ cell development (Carreira‐Rosario et al., 2016). In mammals, myocyte enhancer factor‐2 (Mef2) regulates the expression of miR‐134 which, in turn, downregulates *pum2* transcript to fine‐tune dendrite morphogenesis (Fiore et al., 2009, 2014). In mammals, Mef2 is an activity‐dependent transcription factor that has been implicated to control synapse formation in addition to dendrite morphogenesis (Flavell et al., 2006). Depending on interaction with either positive or negative cofactors, Mef2 can potentiate or repress gene transcription. For example, through an interaction with GATA4, a cardiac‐enriched transcription factor, Mef2 activates the *Nppa* promoter to regulate cardiac development (Morin, Charron, Robitaille, & Nemer, 2000). By contrast, Mef2 forms a complex with class II histone deacetylases (HDACs) to repress gene transcription by deacetylating histones, resulting in chromatin condensation and a reduced accessibility of core transcriptional machinery to promoter regions of target genes (Kao et al., 2001; Lu, McKinsey, Zhang, & Olson, 2000; McKinsey, Zhang, & Olson, 2001).

To identify how transcription of *pum* is regulated, we cloned the promoter region of *dpum* and identified putative binding motifs for 114 transcription factors, including multiple dMef2 elements. A luciferase‐based reporter, driven by the *dpum* promoter, shows that dMef2 is sufficient to transactivate the *dpum* promoter. The magnitude of transactivation varies across the many dMef2 splice variants present in *Drosophila* CNS. Significantly, we also report that dMef2‐mediated transactivation of *dpum* is repressed by p300 (aka Nejire), a histone acetyltransferase (HAT). Unlike dMef2, we show that *p300* expression is directly regulated by neuronal activity and, thus, provide a potential route through which membrane depolarization regulates the expression level of *dpum*.

## 2 MATERIALS AND METHODS

### 2.1 Cloning of expression plasmids

#### 2.1.1 *dpum* promoter

*Pumilio* (*dpum*, CG9755) genomic sequence was obtained from FlyBase (http://flybase.org). Genomic DNA from wild type Canton‐S was extracted in 50 μl extraction buffer (10 mM Tris‐HCl, 1 mM EDTA, 25 mM NaCl and 200 μg/ml proteinase K) with incubation at 37°C for 30 min. *Dpum* promoter constructs were amplified by PCR (Phusion High‐Fidelity DNA Polymerase, New England Biolabs, Hitchin, UK) that consisted of the following in a total volume of 50 μl:20 pmol primers, dNTPs at 0.2 mM and 1X Phusion HF buffer with 1.5 mM Mg^2+^. The forward and reverse primers introduced a *Kpn* I and an *Xho* I sites at the 5’ and 3’ end of promoter respectively. Cycling conditions were: initial denaturation at 98°C for 5 min; 35 cycles of 98°C for 10 s, 55°C for 20 s and 72°C for 2 min 30 s; a final extension step at 72°C for 10 min. The PCR product was digested with *Kpn* I and *Xho* I and ligated into pGL4.23 vector (Promega). The forward and reverse primer sequences are as follows (5’ to 3’): pumA (−2,000 to +1), AATA**GGTACC**CGATGGCTCCGGCGCTGA and pumR: TATT**CTCGAG**GAACATTTAGTGTGACCGCAGCT. A series of deletion constructs for the *dpum* promoter were PCR amplified using forward primers, pumB (−1,434 to +1), AATA**GGTACC**GACCGTCGGCTGGATCCGT, pumC (−578 to +1), AATA**GGTACC**ACATAGCTCGGAAAACGATTTCAAC, pumD (−312 to +1), ATAT**GGTACC**ATGGTTGTATTGATTCTTTATAT and pumE (−189 to +1), ATAT**GGTACC**GGCAACTAGTTAAATGCATTATAG and the reverse primer, pumR.

#### 2.1.2 Amplification of *dmef2* splice variants and *p300*

Total RNA was extracted from the third instar CNS of Canton‐S (mixed sexes). cDNA synthesis was carried out in a total volume of 20 μl using the manufacturer's protocol (RevertAid First‐Strand cDNA Synthesis kit; Thermo Fisher Scientific). *Dmef2* PCR was performed by using forward and reverse primers, which introduced a *Kpn* I and an *Xho* I site, respectively, and ligated to pAc5.1 expression vector (Thermo Fisher Scientific). Fifty‐six plasmids from independent *Escherichia coli* colonies were isolated and sequenced to identify splice variants of *dmef2*. The forward and reverse primer sequences are as follows (5’ to 3’): ATTA**GGTACC**GGATAGGAAATCTGTTGCCATGG and ATTA**CTCGAG**CAGCTCGTGCCGGCTATGT. *p300* (*nejire,* CG15319) was PCR amplified with the primer pairs which introduced *Kpn* I and *Xba* I sites in the 5’ and 3’ end of the open reading frame respectively. PCR product was ligated to pAc5.1 expression vector. The forward and reverse primer sequences are as follows (5’ to 3’): AATA**GGTACC**ATGATGGCCGATCACTTAGACG and AATA**TCTAGA**CTAGAGTCGCTCCACAAACTTG. All clones were checked by sequencing prior to expression analysis.

#### 2.1.3 Identification of transcription factor binding sites

Mouse and human *pum2* promoter sequences (−2,000 to +1) were obtained from the National Centre for Biotechnology Information (NCBI: https://www.ncbi.nlm.nih.gov), mouse: GRCm38:12: 8672314:8674133 and Human: NC_000002.12:c20354428‐20352429. Transcriptional elements and factors were predicted using the TRANSFAC models of MAPPER search engine (Marinescu, Kohane, & Riva, 2005). Mammalian transcription factors associated with human and mouse *pum2* promoters were identified by the Harmonizome search engine (http://amp.pharm.mssm.edu/Harmonizome/).

#### 2.1.4 Luciferase assay

S2R+ cells (10^5^ cells in 100 μl of Schneider's *Drosophila* Medium, Gibco) were treated with dsRNA (1 μg) in a 96‐well plate (Corning^®^ Costar^®^) for 3 hr, followed by cotransfection (Effectene, QIAGEN) of *dpum* promoter‐*firefly* construct and *renilla*‐luciferase reporter (100 ng each) for a further 24 hr. The transfection procedure is as described in the manufacturer's instructions (QIAGEN). The luciferase assay was performed using the dual‐luciferase reporter assay system (Promega). Briefly, 30 μl of transfected S2R+ cells were transferred to a well of a 96‐well white plate (FluoroNunc^™^) and lysed with 30 μl of passive lysis buffer and then 30 μl Luciferase Assay Reagent II was added to measure firefly luciferase activity. This was followed by 30 μl of Stop & Glo^®^ to measure renilla‐luciferase activity. A GENios plate reader (TECAN) was used to measure luminescence. At least five independent transfections of each experiment were performed. Double‐stranded RNA, *dmef2* (BKN27383) and *p300* (BKN21411), were obtained from the Sheffield RNAi Screening Facility (Sheffield, UK).

Luciferase activities of the third instar larvae CNS (mixed sexes) were measured using the Promega Steady‐Glo Luciferase Assay Kit. Briefly, 20 virgin females of *dpum* promoter‐GAL4 line (see below for details) was crossed to five attP24 UAS‐*luciferase* males (Markstein, Pitsouli, Villalta, Celniker, & Perrimon, 2008). Flies carrying the UAS‐*luciferase* transgene alone were used for background controls. Ten third instar larvae CNSs were collected in 100 μl Promega Glo Lysis buffer for each sample, and five independent samples collected for each genotype. CNSs were homogenized, incubated at room temperature for 10 min, centrifuged for 5 min, and supernatant was transferred to a new tube. For luciferase assays, 30 μl of each sample was transferred to a well of a white‐walled 96‐well plate at room temperature, and 30 μl Promega Luciferase reagent was added to each well and plates were incubated in the dark for 10 min. Luminescence was measured with a GENios plate reader (TECAN). The obtained values were normalized to total protein concentration, measured using the Bradford protein assay (Bio‐rad).

Targeted activity‐manipulation was achieved using overexpression of UAS‐*TrpA*, and raised temperature to 29°C for 3 hr. Luciferase activity of *pumC‐GAL4*>*luc*;* TrpA* was normalized to *pumC‐GAL4*>*luc* alone and the measurements at 3 hr compared to 0 hr (set at 1).

#### 2.1.5 Quantitative RT‐PCR

Quantitative RT‐PCR was performed using a SYBR Green I real‐time PCR method (LightCycler^®^ 480 SYBR Green I Master; Roche, Mannheim, Germany) as described in Lin, He, and Baines (2015). For examining *egfp* transcript expression of the *dpum* MiMIC line, 10 third instar larval CNSs were collected in an Eppendorf and treated with or without 10 mM pentylenetetrazol for 1 hr. Total RNA was extracted using the RNeasy microkit (QIAGEN, Hilden, Germany). PCR primers were designed with the aid of LightCycler Probe Design Software 2.0 (v1.0; Roche). Primer sequences (5’ to 3’) used were: *actin‐5C* (CG4027), CTTCTACAATGAGCTGCGT and GAGAGCACAGCCTGGAT; *dmef2*, TTCAAATATCACGCATCACCG and GCTGGCGTACTGGTACA; *p300*, GTTCTGGACTTCCCACG and TACTGGCTCATTTGCATGTAAC; *egfp*, ACGGCAACTACAAGACC and GCTTGTCGGCCATGATATAGA (forward and reverse respectively). The relative gene expression was calculated as the 2^−ΔCt^, where ΔCt was determined by subtracting the average *actin‐5C* Ct value from that of *dmef2*,* p300 or egfp*.

#### 2.1.6 Fly stocks

Transgenic flies were generated using the PhiC31 integrase‐mediated transgenesis system. A *pumC*‐GAL4‐hsp70 fragment was constructed in the pattB vector and microinjected (BestGene Inc., Chino Hills, CA, USA) into an attP‐containing fly stock (Bloomington *Drosophila* Stock Centre stock [BDSC] no. 9748 [RRID: BDSC_9748]: y^1^ w^1118^; PBac{y^+^‐attP‐3B}VK00031). UAS‐*p300* (RRID: BDSC_32573), UAS‐*p300* ^*F2161A*^ (RRID: BDSC_32574), UAS‐*TrpA1(B)* (RRID: BDSC_26263) and *egfp* inserted *dpum* MiMIC‐RMCE (Minos‐mediated integration cassette‐recombination mediated cassette exchange) line (*Mi{PT‐GFSTF.0}pum* ^*MI04825‐GFSTF.0*^; RRID: BDSC_59818) were obtained from Bloomington and UAS‐*p300* ^RNAi^ (stock no. 102885, RRID: FlyBase_FBst0480129) was obtained from the Vienna *Drosophila* Resource Centre. UAS‐*dmef2* was a gift from Dr. Michael Taylor (Cardiff University, Cardiff, UK). The attP24 UAS‐*luciferase* stock was a gift from Dr. Norbert Perrimon (Howard Hughes Medical Institute, Boston, MA, USA).

#### 2.1.7 Yeast two‐hybrid assay

Both *p300* (bait) and *dmef2* (prey) were cloned to pGBKT7 and pGADT7 vector respectively. All yeast strains and plasmids (pGBKT7, pGADT7, pGBKT7‐53, pGADT7‐T and pGBKT7‐Lam) were obtained from Clontech as components of the MATCHMAKER two‐hybrid system 2. The *E. coli* strain *DH5*α was used to clone every shuttle plasmid. Auxotrophic selection plates are Synthetic Dropout (SD) medium supplemented with 0.67% yeast nitrogen base, 0.06% appropriate dropout amino acid mixture and 2% bacto‐agar. The purified bait and prey plasmids were cotransformed into the Y2HGold strain using a lithium‐acetate method according to the manufacturer's instructions (Clontech) and were then cultured on SD/‐Trp/‐Leu agar plates. Approximately 2 mm of Y2HGold transformants were transferred to SD/‐Trp/‐Leu/‐His/‐Ade and SD/‐Trp/‐Leu/‐His/‐Ade/X‐α‐gal/Aureobasidin A plates at 30°C for 2~3 days. The host *Saccharomyces cerevisiae* strain Y2HGold genotype is as follows: *MATa*,* trp1‐901*,* leu2‐3*,* 112*,* ura3‐52*,* his3‐200*,* gal4Δ*,* gal80Δ*,* LYS2 : : GAL1*_*UAS*_ *–Gal1*_*TATA*_ *–His3*,* GAL2*_*UAS*_ *–Gal2*_*TATA*_ *–Ade2 URA3 : : MEL1*_*UAS*_ *–MEL1*_*TATA*_ *AUR1‐C MEL1*. In mammals, protein–protein interaction between the cysteine–histidine‐rich region 3 (CH3) domains containing C‐terminus of p300 and Mef2A/Mef2C (through MASD/Mef2 domains) has been reported (De Luca et al., 2003; Sartorelli, Huang, Hamamori, & Kedes, 1997). Therefore, p300_301‐3276_ (2976 amino acids, from 301th to the end of C‐terminus (3276th)) and full‐length dMef2 were cloned. p300 bait, BD‐*p300* DNA fragment was released from *p300*/pAc5.1 using *Nde* I and *Xba* I (filling the sticky end to blunt end with Klenow) and ligating into pGBKT7 (*Nde* I and *Sma* I). dMef2 prey, AD‐*dmef2* DNA fragment was released from *dmef2(VI)*/pAc5.1 or *dmef2(VII)*/pAc5.1 using *Kpn* I (filling the sticky end to blunt end with Klenow) and *Xho* I and ligating into pGADT7 (*Bam*H I [filling the sticky end to blunt end with Klenow] and *Xho* I).

### 2.2 Statistics

Statistical significance between group means was assessed using either a Student's *t* test (where a single experimental group is compared to a single control group and *p*‐values are presented two‐sided) or a one‐way ANOVA followed by Bonferroni's post hoc test (multiple experimental groups). Data are presented as mean ± standard deviation (*SD*).

## 3 RESULTS AND STATISTICAL ANALYSES

### 3.1 Identification of the *Pumilio* promoter region

**Figure 1:**
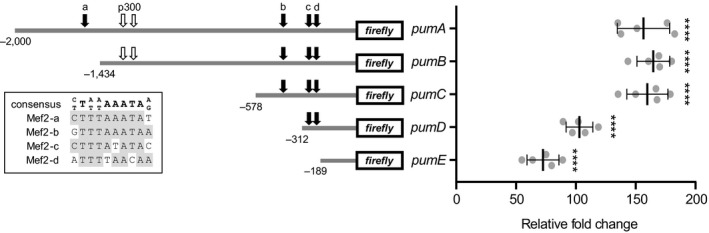
Characterization of *dpum* promoter activity. Activity analysis, by luciferase assays, of constructs bearing defined regions of a putative 2‐kb *dpum* promoter that was placed upstream of *firefly*‐luciferase (*FF*). These regions are termed *pumA*,* pumB*,* pumC*,* pumD* and *pumE* respectively (−2,000, −1,434, −578, −312 and −189 to +1: transcription initiation marked as +1). Constructs were transiently cotransfected, together with a *renilla*‐luciferase (*Ren*, a loading control, driven by *actin* promoter), in S2R+ cells for 24 hr and *pum:FF* to *Ren* ratio was calculated. *PumA*,* pumB*,* pumC*,* pumD* and *pumE:FF* resulted in 156.5 ± 21.9‐, 164.8 ± 13.7‐, 159.8 ± 17.2‐, 103 ± 11.1‐ and 72.5 ± 13.4‐fold increase in FF activity compared to transfection of cells with empty vector (FF to Ren ratio was set at 1; *n *=* *5 independent transfections). Four putative Mef2 binding sites (Mef2‐a, ‐b, ‐c and ‐d) with a consensus sequence (C/T)T(A/T)(A/T)AAATA(A/G) were identified (black arrows) and each binding sequence is shown in the inset (identical nucleotides shown in grey boxes). White arrows indicate two potential p300 binding sites. The location of putative transcriptional binding motifs, Sp1, TBP, C/EBP, Oct‐1, Mef2, MADS‐A/B, Hb, NF‐kappaB, TCF, CREB and SRF within the *dpum* 2‐kb promoter region and the initiation of each *dpum* promoter fragment, *pumA‐E*, are indicated in Supporting Information Figure S1. The detailed binding motifs identified within the 2‐kb promoter region of *dpum*, mouse *pum2* and human *pum2* are listed in Supporting Information Tables S1–S3 respectively. Data are presented as mean ± *SD* ****p ≤* *0.0001 (ANOVA with Bonferroni's post hoc)

To analyse transcriptional regulation of *dpum*, we identified a putative *dpum* promoter region. A 2‐kb region upstream of the transcription start site was targeted as a potential location. Interrogation of transcription factor databases (TRANSFAC model, MAPPER; Marinescu et al., 2005), identified putative binding motifs for 114 transcription factors within the region −2,000 to +1 (transcription initiation marked as +1, motifs listed in Supporting Information Table S1). Putative transcriptional binding motifs include: Sp1, TBP, C/EBP, Oct‐1, Mef2, MADS‐A/B, Hb, NF‐kappaB, TCF, CREB and SRF (Supporting Information Figure S1). To test for function of the *dpum* promoter region, this 2‐kb fragment, (−2,000 to +1, termed *pumA*) was placed upstream of *firefly*‐luciferase (*FF*) and transiently transfected in S2R+ cells. After 24 hr, *pumA:FF* resulted in a 156.5 ± 21.9‐fold increase in FF activity compared to transfection of cells with empty vector (set at 1, Figure 1, *p* = 2.5 × 10^−7^).

To evaluate a minimal promoter region of *dpum*, we generated a series of deletion constructs in addition to *pumA*:* pumB* (−1,434 to +1), *pumC* (−578 to +1), *pumD* (−312 to +1) and *pumE* (−189 to +1; Figure 1). Compared to *pumA:FF*,* pumB:FF* and *pumC:FF* resulted in a similar increase in FF expression (164.8 ± 13.7 and 159.8 ± 17.2‐fold, *p *=* *4 × 10^−9^ and 3.2 × 10^−8^, respectively), while *pumD:FF* and *pumE:FF* showed lower, but still significant, increases (103 ± 11.1 and 72.5 ± 13.4‐fold increase, *p *=* *3.2 × 10^−8^ and 2.2 × 10^−6^ respectively; Figure 1). Thus, it would seem that multiple elements contained within the 2‐kb region are capable of increasing *dpum* expression.

### 3.2 dMef2 transactivates the *Pumilio* promoter

Mef2 is reported to reduce *pum2* transcript post‐transcriptionally through increased expression of miR‐134 (Fiore et al., 2009). However, our analysis of the 2‐kb *dpum* promoter identified four putative Mef2 binding sites with the consensus sequence (C/T)T(A/T)(A/T)AAATA(A/G) (Gossett, Kelvin, Sternberg, & Olson, 1989; Figure 1). These four Mef2 elements (termed Mef2‐a, ‐b, ‐c and ‐d) are located at ‐1561, ‐423, ‐298 and ‐214 bp respectively (Figure 1). This high number of sites is indicative of direct regulatory effect. To test how Mef2 influences *dpum* expression, we used *Drosophila mef2* (*dmef2*) dsRNA (specific for all variants) to knockdown endogenous expression in S2R+ cells that coexpressed the *pumA:FF* reporter. This was sufficient to significantly reduce *pumA* promoter activity (154.5 ± 13.1 vs. 113.2 ± 15.1 fold increase, control vs. *dmef2* dsRNA, *p *=* *0.006) indicative that *dpum* expression is endogenously regulated, at least in part, by dMef2.

**Figure 2:**
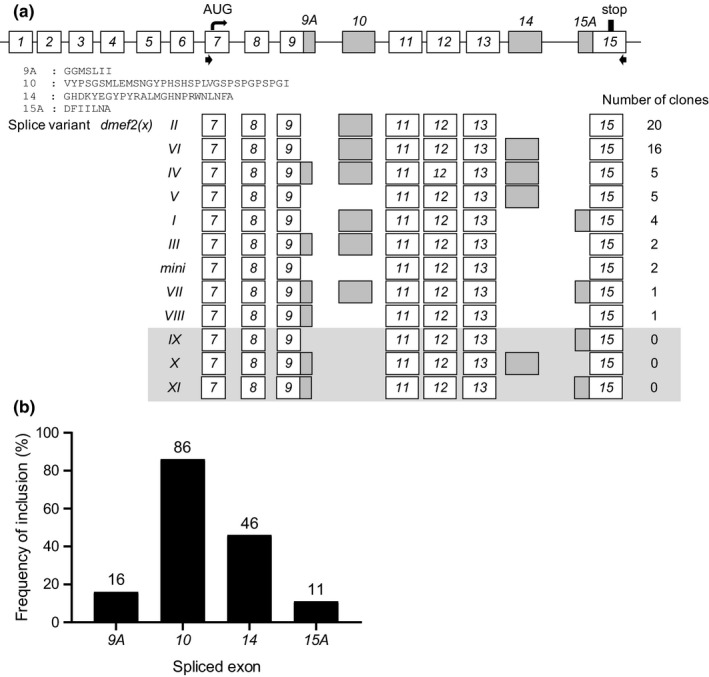
Characterization of splice variants of *dmef2* isolated from the third instar larval central nervous system. (a) Schematic of the *dmef2* gene structure. *Dmef2* contains 15 exons with exon *10* and *14* being alternatively spliced, while exons *9* and *15* contain cryptic splice sites and generate cassettes 9A and 15A respectively. The amino acid sequences of exons 9A, 10, 14 and 15A are shown. Black arrows indicate the location of primer pairs used to amplify the open reading frame of *dmef2*. Exon usage of splice variants, termed *dmef2(I‐VIII)* and *dmef2(mini*), and the frequency of clones are indicated. The clones, *dmef2(IX–XI)*, in the shaded box were not found in the third instar CNS, but are theoretically possible. (b) Analysis of exon usage across the identified *dmef2* splice variants shows that exon *10* is present at highest abundance (86%) followed by exon *14* (46%)

*Dmef2* is encoded by a single gene (Lilly, Galewsky, Firulli, Schulz, & Olson, 1994; Nguyen, Bodmer, Abmayr, McDermott, & Spoerel, 1994; Taylor, Beatty, Hunter, & Baylies, 1995) and contains 15 exons. Exons *10* and *14* are alternatively spliced, while exons *9* and *15* contain cryptic splice sites and generate cassettes 9A and 15A respectively (Figure 2a). To identify the most common splice isoforms of *dmef2* transcripts present in the CNS of third instar *Drosophila* larvae, RT‐PCR was used to isolate and clone 56 complete open reading frames (ORFs). Comparison of exon composition of clones revealed nine unique splice variants. Isoforms *dmef2(I–IV)* were previously identified (Gunthorpe, Beatty, & Taylor, 1999; Taylor et al., 1995), while *dmef2(V–VIII)* and *dmef2(mini)* are novel. Analysis of the ORFs showed that *dmef2(II)* and *dmef2(VI)* are present at highest frequency (Figure 2a). Analysis of exon usage across all splice sites show that exon *10* is present at highest abundance followed by exon *14* (Figure 2b).

**Figure 3:**
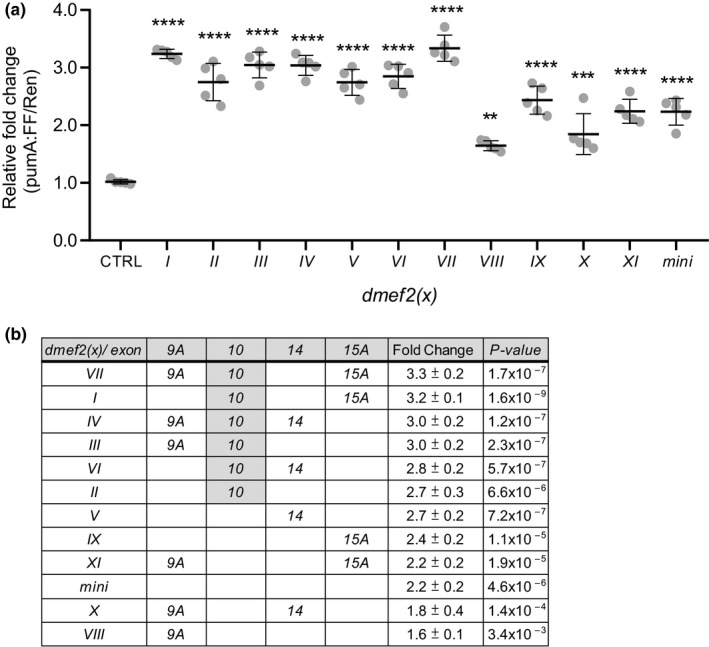
dMef2 splicing variants transactivate the *dpum* promoter. (a) Expression of *pumA* promoter:*firefly‐*luciferase (*pumA:FF*) reporter with individual *dmef2* splice variants (*dmef2(x)*) and a *renilla*‐luciferase gene (*Ren*, loading control, driven by *actin* promoter) in S2R+ cells. Expression of *dmef2* variants resulted in a 1.6 ± 0.1 to 3.3 ± 0.2‐fold increase in luciferase activity compared to control (CTRL, no *dmef2* expression, set at 1; *n *=* *5 independent transfections). (b) Effectiveness of each splice variant ranked highest to lowest. Exon *10* containing variants, *dmef2(I, II, III, IV, VI and VII),* transactivated the *dpum* promoter to a greater level than variants lacking this exon: *dmef2(V, VIII, IX, X, XI and mini)*. Data are presented as mean ± *SD*. **p ≤* *0.01 and ****p ≤* *0.0001 (ANOVA with Bonferroni's post hoc)

To gain better understanding of how individual dMef2 splice variants transactivate *pumA:FF* activity, we constructed another three splice variants (although not found in our CNS analysis, these variants are theoretically possible), *dmef2(IX–XI)* (Figure 2a). The expression of individual *dmef2* variants, in S2R+ cells, resulted in a 1.6 ± 0.1‐ to 3.3 ± 0.2‐fold increase in FF expression compared to control (only intrinsic *dmef2* expression, set at 1; Figure 3a). This level of change mirrors previous reports of transgenic MEF2‐transactivation of other transcripts (ranging from ~2.5‐ to 2.9‐fold; Lyons, Schwarz, & West, 2012; Wang, Wang, Chen, & Sun, 2017). Sorting splice variants by fold change formed a clear group of *dmef2* isoforms that contain exon *10* (Figure 3b). Exon *10* is contained within *dmef2 (I*,* II*,* III*,* IV*,* VI* and *VII)* that, collectively, transactivated the *dpum* promoter to a greater level than variants lacking this exon: *dmef2 (V*,* VIII*,* IX*,* X*,* XI* and *mini*). Comparing identical *dmef2* variants that differ only by the inclusion of exon *10* (e.g. *dmef2 (I)* vs. (*IX)*; (*II)* vs. (*mini)*; (*III)* vs. (*VIII)*; (*IV)* vs. (*X)* and (*VII)* vs. (*XI), p = *2.9 × 10^−5^, 0.005, 1.2 × 10^−6^, 0.0002 and 2.5 × 10^−5^, *t* test, respectively) confirms that inclusion of this exon results in further significant transactivation.

**Figure 4:**
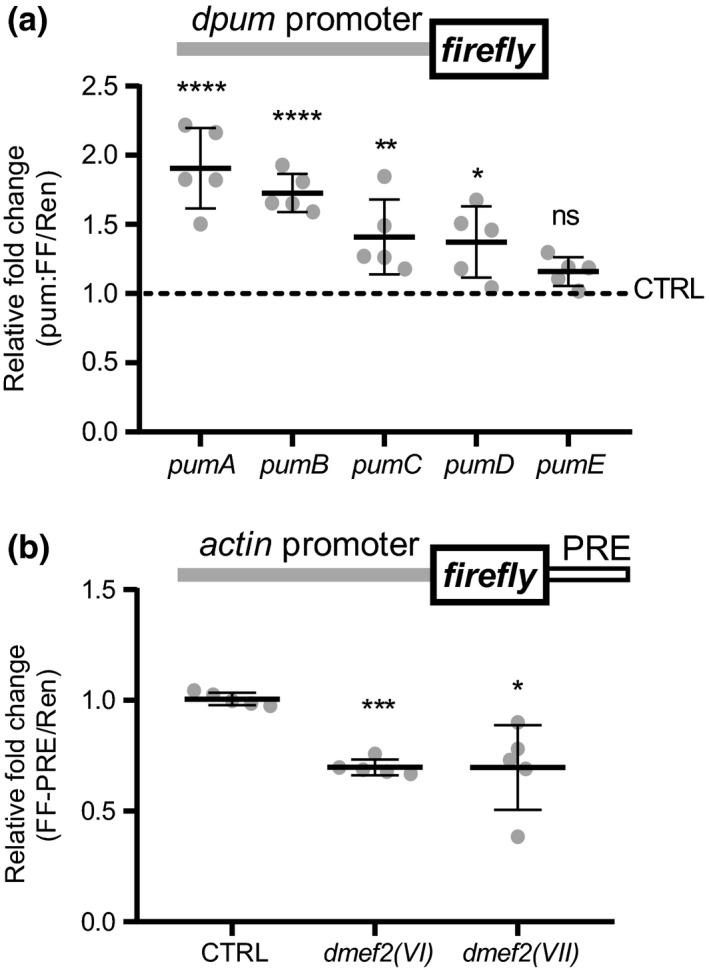
dMef2 is sufficient to modify *dpum* promoter transactivation and dPum protein activity. (a) *dpum* promoter:*firefly*‐luciferase (*pum*:*FF*) constructs, containing different numbers of dMef2 binding motifs (*pumA‐E*, 4, 3, 3, 2 and 0, respectively), were cotransfected with *dmef2(VII)* and a *renilla*‐luciferase gene (*Ren*, loading control, driven by *actin* promoter) in S2R+ cells. Expression of *dmef2* resulted in 1.9 ± 0.3‐, 1.7 ± 0.1‐, 1.4 ± 0.3‐, 1.4 ± 0.3‐ and 1.2 ± 0.1‐fold increase in promoter activity (*pumA‐E*, respectively) compared to control (set at 1: each construct expressed in the absence of *dmef2(VII))* (*n *=* *5 independent transfections). (b) dPum protein activity can be measured using an *actin* promoter driven *firefly*‐luciferase (FF) reporter gene containing Pumilio Response Elements (PRE) in the 3’ UTR (FF‐PRE). Increased dPum is sufficient, through binding the PREs and inhibiting translation, to reduce FF activity. An identical *renilla*‐luciferase (Ren) reporter, but lacking the PRE sites (and thus not affected by dPum), is coexpressed to allow ratiometric determination of activity. Expression of *dmef2(VI)* or *dmef2(VII)* isoforms in S2R+ cells, that express both reporters, significantly reduces the ratio of FF‐PRE/Ren to 0.7 ± 0.04 and 0.7 ± 0.21 respectively (control [CTRL], no *dmef2* expression, set as 1; *n *=* *5 independent transfections). Data are presented as mean ± *SD*. *p ≤* *0.05, **p ≤* *0.01, ***p ≤* *0.001 and ****p ≤* *0.0001. ns: not significant. (ANOVA with Bonferroni's post hoc)

To confirm dMef2 transactivation of *dpum*, we compared FF activity of each *dpum* promoter construct (*pumA* to *E*) following *dmef2*(*VII*) overexpression (the variant showing the strongest transactivation). Promoter fragments *pumA–E* contain 4, 3, 3, 2 and 0 predicted Mef2 binding elements respectively (Figure 1). Overexpression of *dmef2* resulted in 1.9 ± 0.3‐, 1.7 ± 0.1‐, 1.4 ± 0.3‐ and 1.4 ± 0.3‐fold increase in promoter activity (*pumA–D*,* p *=* *1.2 × 10^−6^, 4.1 × 10^−6^, 0.004, 0.05, respectively) compared to control (no *dmef2* expression, set at 1) while, as predicted, *pumE* (lacking an Mef2 binding motif) showed no change (1.2 ± 0.1, *p *>* *0.05; Figure 4a).

### 3.3 dMef2 regulates Pumilio expression level

To confirm observations of transcriptional regulation of *dpum* by dMef2, we used a previously developed dPum protein activity monitor to determine effect to dPum protein level (Lin et al., 2017). Essentially, we constructed an *actin* promoter driven *firefly*‐luciferase reporter gene (*FF*‐PRE), containing PREs (Pumilio Response Elements), in the 3’ UTR. Increased dPum is sufficient, through binding the PREs and inhibiting translation, to reduce FF activity. An identical *renilla*‐luciferase (*Ren*) reporter, lacking the PRE sites (and thus not affected by dPum), is coexpressed to allow ratiometric determination of activity (to compensate for batch differences between construct expression). Although this is an indirect measurement, it is currently the best monitor of dPum protein activity, because of the poor performance of commercially available anti‐Pum antibodies for Western Blotting in *Drosophila* (our unpublished data). Overexpression of *dmef2(VI)* or *dmef2(VII)* isoforms in S2R+ cells, that express both reporters, significantly reduces the ratio of *FF*‐PRE/*Ren* to 0.7 ± 0.04 and 0.7 ± 0.21, respectively (control, no *dmef2* expression, set as 1, *p *=* *0.00013 and 0.03 respectively, Figure 4b). We conclude that increasing *dmef2* is sufficient to increase dPum protein activity, in addition to transcript.

### 3.4 p300 suppresses dMef2‐mediated *Pumilio* transactivation

Our prior work has shown levels of dPum and rPum2 (in fly and rat, respectively) are sensitive to neuronal activity: increasing as levels of synaptic excitation increase (Driscoll et al., 2013; Mee, Pym, Moffat, & Baines, 2004). It was expected, therefore, that *dmef2* would show activity‐dependent transcription. Similar to mammals, ingestion of the proconvulsant PTX by larvae is sufficient to increase synaptic excitation and induce a seizure‐like state (Stilwell, Saraswati, Littleton, & Chouinard, 2006). Therefore, we performed RT‐qPCR to examine *dmef2* transcript expression in the CNS taken from PTX‐fed larvae. We did not, however, observe a significant fold‐change (0.97 ± 0.06, *n *=* *5, *p *>* *0.05) compared to vehicle control (set at 1). This lack of effect is indicative that the expression of *dmef2*, in *Drosophila*, is not activity dependent. We are not, however, able to rule out activity‐dependent post‐transcriptional and/or post‐translational modifications of dMef2 which may, in turn, influence the expression or activity of dPum.

p300 is a reported coregulator of Mef2A in mammals (De Luca et al., 2003) and we identify potential binding sites (GGGAG) for this protein in the *dpum* promoter (Chen & Hung, 1997; Rikitake & Moran, 1992), (see Figure 1). A previous RNA‐seq analysis, between isolated CNS taken from WT larvae and WT larvae fed PTX, identified *Drosophila p300* to be significantly downregulated by enhanced synaptic excitation (214 ± 10 vs. 163 ± 2 counts per million, WT vs. WT fed PTX, *n *=* *3, *p *=* *0.001; Lin et al., 2017). To confirm this observation, we performed RT‐qPCR to examine *p300* transcript abundance in CNS taken from PTX‐fed WT larvae: *p300* transcript expression is downregulated to 0.87 ± 0.04 (*n *=* *5, *p *=* *0.0026) compared to vehicle control (set at 1).

**Figure 5:**
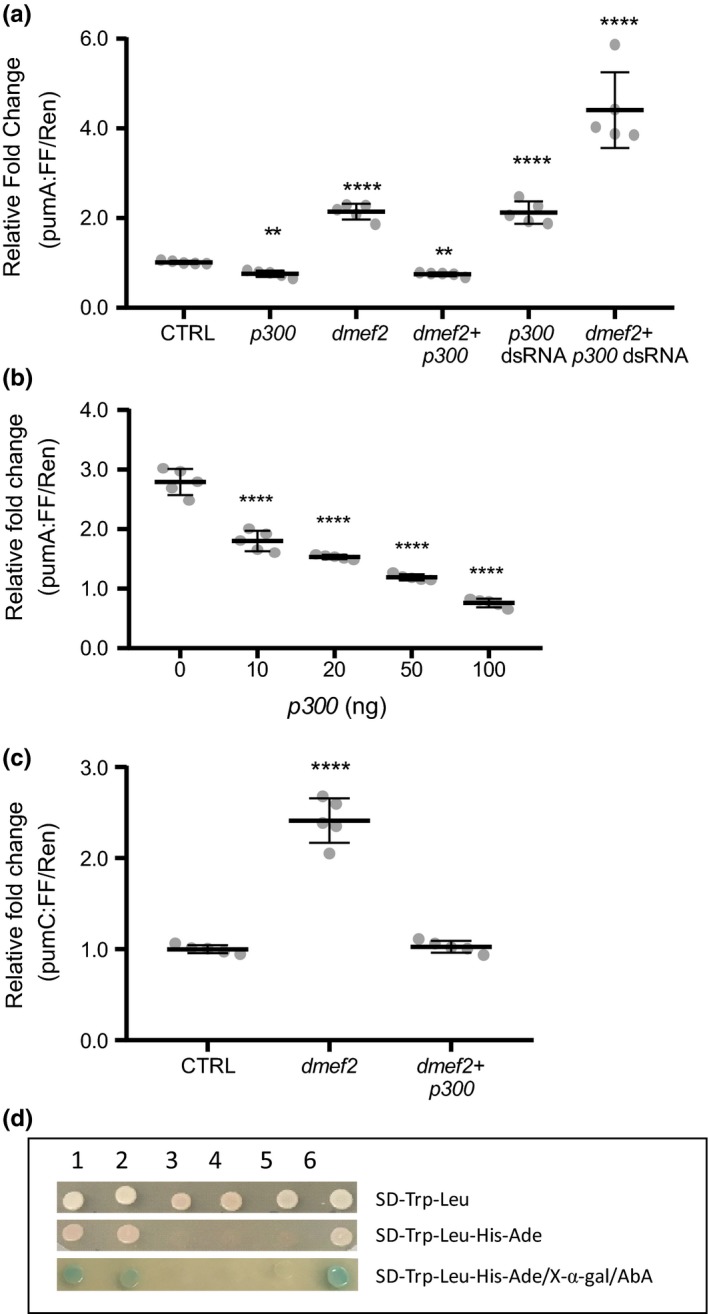
p300 represses dMef2‐mediated transactivation of the *dpum* promoter. (a) The *pumA* promoter:*firefly*‐luciferase (*pumA*:*FF*) reporter was cotransfected with *p300* and a *renilla*‐luciferase gene (*Ren*, loading control, driven by *actin* promoter) in S2R+ cells. Expression of *p300* reduced *pumA* promoter activity to 0.76 ± 0.07, (control [CTRL], pumA:FF/Ren alone set at 1). By comparison, the expression of *dmef2(VII)* resulted in a 2.14 ± 0.18‐fold increase in luciferase activity compared to control. The activity of *dmef2(VII)* is abolished when coexpressed with *p300* (0.75 ± 0.04). Pre‐treatment of S2R+ cells with *p300* dsRNA enhanced *pumA* promoter activity to 2.12 ± 0.22. Overexpression of *dmef2*, in the presence of *p300* dsRNA, further increased *pumA* promoter activity to 4.41 ± 0.74 (*n *=* *5 independent transfections). (b) Co‐transfection of *pumA:FF* with increasing doses of *p300* showed a clear dose‐dependent suppression of dMef2(VII)‐mediated transactivation (2.79 ± 0.22, 1.8 ± 0.17, 1.53 ± 0.03, 1.19 ± 0.05 and 0.76 ± 0.07, *p300* plasmid: 0, 10, 20, 50 and 100 ng, respectively, pumA:FF/Ren alone set at 1; *n *=* *5 independent transfections). (c) Co‐transfection of *pumC*:*FF* (which lacks consensus p300 binding sequences) with *dmef2(VII)* resulted in a 2.41 ± 0.2‐fold increase in luciferase activity compared to control (CTRL, pumC:FF/Ren alone set at 1). This enhancement is abolished when coexpressed with *p300* (1.03 ± 0.06, *n *=* *5 independent transfections). (d) Yeast two‐hybrid assay shows a protein–protein interaction between dMef2 and p300. The bait plasmid pGBKT7‐*p300* (BD *‐p300*) and the prey pGADT7‐*dmef2* (AD *‐dmef2(VI)* or AD‐*dmef2(VII)*) were cotransformed into a Y2HGold yeast strain. Coexpression of 1. BD‐p300/AD‐dMef2(VI) or 2. BD‐p300/AD‐dMef2(VII) resulted in activation of all four GAL4‐responsive markers, *HIS3*,*ADE2*,*AUR1‐C* and *MEL1* reporters in Y2HGold. The negative control groups, 3. BD‐lam/AD‐dMef2(VI), 4. BD‐lam/AD‐dMef2(VII) and 5. BD‐p300/AD‐T, were not able to activate reporter gene expression in Y2HGold. 6. BD‐p53/AD‐T was used as a positive control. AbA: Aureobasidin A, BD: GAL4 DNA binding domain, AD: GAL4 activation domain, SD: synthetic dropout selective media, Lam: Lamin C, T: large T antigen. Data are presented as mean ± *SD*. **p ≤* *0.01 and ****p ≤* *0.0001 (ANOVA with Bonferroni's post hoc). [Colour figure can be viewed at wileyonlinelibrary.com]

To test how p300 contributes to transcriptional regulation of *dpum* expression, we coexpressed *p300* and *pumA:FF* in S2R+ cells. Overexpression of *p300* was sufficient to reduce *pumA* promoter activity to 0.76 ± 0.07 (*pumA:FF* alone set at 1, *n *=* *5, *p *=* *0.005). Moreover, dMef2‐mediated activation of *pumA:FF* is abolished when coexpressed with *p300* (0.75 ± 0.04, *n *=* *5, *p *=* *0.0034; Figure 5a). Cotransfection with varied doses of *p300* showed a clear dose‐dependent suppression of dMef2‐mediated transactivation (2.79 ± 0.22, 1.8 ± 0.17, 1.53 ± 0.03, 1.19 ± 0.05 and 0.76 ± 0.07, *p300* plasmid: 0, 10, 20, 50 and 100 ng, respectively, *p *=* *4.2 × 10^−15^; Figure 5b). Pre‐treatment of S2R+ cells with *p300* dsRNA enhanced *pumA:FF* promoter activity to 2.12 ± 0.22 (*n *=* *5, *p *=* *0.0001; Figure 5a). Finally, overexpression of *dmef2*, in the presence of *p300* dsRNA, further increased *pumA:FF* activity to 4.41 ± 0.74 (*n *=* *5, *p *=* *0.0001; Figure 5a). Collectively, these data suggest that p300 negatively regulates the *dpum* promoter. However, it does not discriminate whether p300 acts through direct binding to the *dpum* promoter.

To test whether binding of p300 to the *dpum* promoter is required for repression of dMef2‐dependent *dpum* transactivation, we expressed *p300* and *dmef2* with *pumC:FF*, which lacks consensus p300 binding sequences (see Figure 1). While the expression of dMef2 enhanced *pumC:FF* activity (2.41 ± 0.2, *pumC:FF* alone set at 1, *n *=* *5, *p *=* *1.4 × 10^−6^), coexpression of *p300* was still sufficient to significantly reduce dMef2‐mediated transactivation (1.03 ± 0.06, *n *=* *5, *p *>* *0.05, Figure 5c). Thus, direct binding of p300 to the *dpum* promoter is seemingly not required for repression of dMef2‐mediated *dpum* transactivation, indicative of a protein–protein interaction between p300 and dMef2. To demonstrate a physical interaction between p300 and dMef2, we performed a yeast two‐hybrid assay. The bait plasmid pGBKT7‐*p300* (BD*‐p300*) and the prey pGADT7‐*dmef2* (AD*‐dmef2(VI)* or AD‐*dmef2(VII)*) were cotransformed into a Y2HGold yeast strain. Coexpression of BD‐p300 with AD‐dMef2(VI) or AD‐dMef2(VII) resulted in activation of all four GAL4‐responsive markers, *HIS3*,* ADE2*,* AUR1‐C* and *MEL1* reporters in Y2HGold. Conversely, coexpression of control groups, BD‐p300/AD‐T (large T antigen) or AD‐dMef2/BD‐Lam (Lamin C), was not able to activate reporter gene expression, indicative that the autoactivation of BD‐p300 and AD‐dMef2 were negligible (Figure 5d). This result strongly suggests that p300 directly binds dMef2.

### 3.5 p300 represses *Pumilio* promoter activity in vivo

To measure *dpum* promoter activity in vivo, we generated a transgenic *pumC‐GAL4* fly and mated it with UAS‐*luciferase* (UAS‐*luc*; Markstein et al., 2008). The resultant third instar larval CNS, expressing *pumC‐GAL4*>*luc* showed a significant increase (2.6 ± 0.6‐fold) in luc activity compared to control (UAS‐*luc* line, set at 1, *p *=* *0.0005, *t* test, *n *=* *5). To test how the *pumC* promoter responds to increased synaptic excitation, we established a stable line of *pumC‐GAL4*>*luc* and raised larvae on food containing PTX (1 μg/ml). This resulted in a 1.9 ± 0.4‐fold increase in luc compared to the vehicle control (i.e. no PTX, set at 1, *p *=* *0.009, *t* test, *n *=* *5). To restrict activity‐manipulation to only *dpum* expressing cells, we crossed *pumC‐GAL4*>*luc* with UAS‐*TrpA* and raised the ambient temperature to 29°C (activating the TrpA channel). This also resulted in a 2.7 ± 1.3‐fold increase in luc expression compared to a 25°C control (set at 1, *p *=* *0.004, *t* test, *n *=* *15). To further confirm activity dependence of the *dpum* promoter, we exploited an *egfp*‐inserted *dpum* MiMIC‐RMCE (Minos‐mediated integration cassette‐recombination mediated cassette exchange) line. This line has *egfp* inserted within the *dpum* locus. Exposure of isolated third instar larval CNS to the proconvulsant pentylenetetrazole (PTZ; 10 mM) for 1 hr resulted in a 2.2 ± 1.3‐fold increase in *egfp* transcript expression compared to untreated larvae (set at 1, *p *=* *0.05, *t* test, *n *=* *8). For this acute treatment on isolated CNS, we used PTZ (a water‐soluble GABA_A_ receptor inhibitor) rather than PTX (required to be dissolved in DMSO) to avoid a confounding effect of the vehicle when using isolated CNS (but not observed with whole larvae). These results not only confirm our previous observations that *dpum* expression is regulated by membrane depolarization (Mee et al., 2004), but transfers our capability to measure *dpum* promoter activity to in vivo.

**Figure 6:**
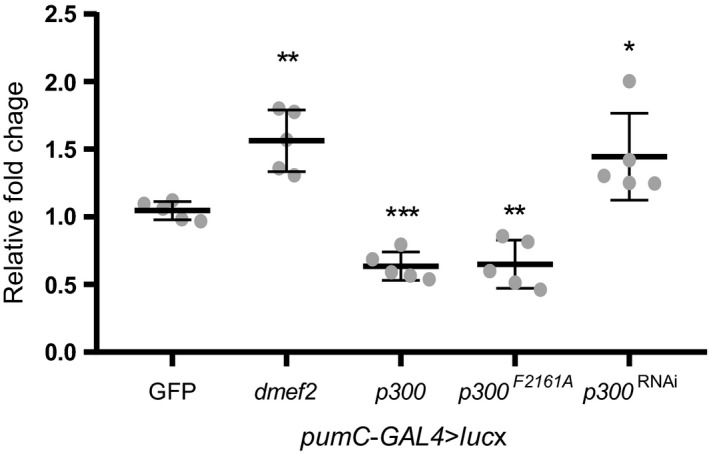
dMef2 and p300 regulate *dpum* promoter activity in vivo. Expression of *dmef2, p300* or *p300* ^*F2161A*^, respectively, resulted in a 1.6 ± 0.2, 0.6 ± 0.1 and 0.6 ± 0.2‐fold change in luciferase (luc) activity compared to control (*pumC‐GAL4>luc* cross with UAS‐*GFP* (*GFP*), set at 1). Knockdown of *p300* expression, achieved by crossing UAS‐*p300* ^RNA^ ^i^ (*p300* ^*RNA*^ ^*i*^), resulted in a 1.4 ± 0.3‐fold increase in luc activity (*n *=* *5 independent sample collections). Data are presented as mean ± *SD*. *p ≤* *0.05, **p ≤* *0.01 and ***p ≤* *0.001 (ANOVA with Bonferroni's post hoc)

Overexpression of *dmef2*, by crossing *pumC‐GAL4*>*luc* with UAS‐*dmef2*, resulted in a 1.6 ± 0.2‐fold increase in luc expression compared to *pumC‐GAL4*>*luc* crossed with control UAS‐*GFP* (set at 1, *p *=* *0.003 *n *=* *5, Figure 6). This confirms that the *pumC* promoter is transactivated by dMef2 in vivo. To validate our observation that p300 represses *dpum* expression, we also crossed *pumC‐GAL4*>*luc* with UAS‐*p300* or UAS‐*p300* ^RNAi^. Overexpression of *p300* resulted in a 0.6 ± 0.1‐fold reduction in luc activity (*p *=* *0.0004, *n *=* *5), while *p300* knockdown increased luc expression by 1.4 ± 0.3‐fold (*p *=* *0.03, *n *=* *5; Figure 6). p300 contains histone acetyltransferase (HAT) activity (Ogryzko, Schiltz, Russanova, Howard, & Nakatani, 1996). To test if HAT activity is required for the repression of *dpum* transactivation, we crossed *pumC‐GAL4*>*luc* with UAS‐*p300* ^*F2161A*^, a mutant with abolished HAT activity (Ludlam et al., 2002). Overexpression of *p300* ^*F2161A*^ still resulted in a 0.6 ± 0.2‐fold reduction in luc activity (*p *=* *0.003, *n *=* *5; Figure 6), indicating that p300‐dependent repression of *dpum* expression is independent of HAT activity.

Finally, to test the capability of p300 to inhibit *dpum* promoter activation under conditions of enhanced synaptic activity, we raised larvae on food containing PTX (1 μg/ml). PTX‐fed controls (*pumC‐GAL4*>*luc*; >*GFP*) resulted in an expected 1.9 ± 0.8‐fold increase in luc activity compared to vehicle control (set at 1, *p *=* *0.01, *n *=* *10). This was reduced to 0.9 ± 0.03 when *p300* was coexpressed (*pumC‐GAL4*>*luc*; >*p300*,* p *>* *0.05, *n *=* *10). This result demonstrates that overexpression of *p300* is sufficient to prevent PTX‐induced transactivation of *dpum* promoter activity. Thus, we validate, in vivo*,* that dMef2 is sufficient to transactivate the *dpum* promoter and that this activity is negatively regulated by p300.

## 4 DISCUSSION

Pumilio is a well characterized RNA‐binding protein that, among its many reported functions, is a key regulator of neuronal firing‐rate homeostasis that maintains stability of neuronal circuits (Giachello & Baines, 2017; Mee et al., 2004; Muraro et al., 2008). Despite this critical role, little is known concerning how Pum levels are regulated. Its involvement with neuronal homeostasis suggests that *pum* transcription will be governed by an activity‐dependent process (Mee et al., 2004). In this study, we characterized the *dpum* promoter region and identified both Mef2 and p300 to be part of an activity‐dependent regulatory mechanism. The complete regulatory mechanism is, however, likely to be more complicated based on our identification of binding motifs for 114 putative transcription factors within a 2‐kb region upstream of the *dpum* transcription start site. A series of deletion constructs show that *pumA*,* pumB* and *pumC* promoters (composed of 2,000, 1,434 and 578 nucleotides, respectively) exhibit promotor activity, while *pumD* and *pumE* (consist of 312 and 189 nucleotides, respectively) result in reduced, but still significant, activity. The *pumE* promoter, the shortest construct we tested, contains binding motifs for 15 transcription factors (e.g., Pbx‐1, BR‐C Z4, Zen and Lhx3a) and is, as we show, still capable of driving *FF* expression in S2R+ cells. Further truncations will be required to identify the minimal promoter region for *dpum*.

We identify four dMef2 binding motifs within a 2‐kb putative *dpum* promoter region and, moreover, show that manipulating *dmef2* expression is sufficient to influence promoter activity. Comparison with genes known to be transactivated by dMef2 (e.g., *myoD*,* inflated, mir‐1, tubulin60D* and others) identifies, on average, 5 ± 1.6 Mef2 binding motifs (analysis of eight genes ± SD). By comparison, analysis of a similar number of genes with no reported regulation by Mef2 (e.g., *tailup*,* cry*,* lim3*,* eve*,* shaker* and three others) identifies 3.2 ± 1.4 binding motifs per gene (*p *=* *0.009, unpaired *t* test, Lin and Baines, unpublished data). This difference strongly suggests that *dpum* expression is regulated, at least in part, by dMef2.

*Dmef2*, which is encoded by a single gene, contains 15 exons. Of these exons, four are subject to alternative splicing and generate at least 12 splice variants (*dmef2(I*–*XI)* and *dmef2(mini)*). All splice isoforms transactivate the *dpum* promoter and, notably, exon 10‐containing isoforms result in greater activation compared to variants lacking this exon. This differential activity may be indicative that the level of *dpum* transactivation can be fine‐tuned through altering of dMef2 isoform expression, a possibility that will require future investigation. In muscle, by contrast, it is the expression level of dMef2, rather than isoform expression, which is seemingly more important for cellular differentiation (Gunthorpe et al., 1999). This conclusion was reached, however, without consideration of exon 10 lacking isoforms, because splicing of exon *10* was not previously observed (Gunthorpe et al., 1999; Taylor et al., 1995). Our bioinformatics shows that Mef2 binding sites are conserved in the mammalian (e.g. human and mouse) *pum2* promoter region. Interrogation using the Harmonizome search engine followed by ChIP‐X enrichment analysis (ChEA) of transcription factor targets database, identifies 23 transcription factors, including p300 and Mef2A, associate with the *pum2* promoter region (Lachmann et al., 2010; Rouillard et al., 2016). A genome‐wide tiling array (ChIP‐Chip) analysis similarly identifies *dpum* as a target of dMef2 (Sivachenko, Li, Abruzzi, & Rosbash, 2013). Our analysis of the promoter region (−2,000 to +1, set transcription initiation at +1), in both mouse and human *pum2*, identifies 5 and 6 Mef2 elements, respectively (Supporting Information Tables S1 and S2). Thus, it seems likely that Mef2 is a direct regulator of *pum* transcription in both insects and mammals. This extends the activity of this transcription factor in addition to its reported inhibitory control of *pum2* transcript abundance via upregulation of miR‐134 (Fiore et al., 2009, 2014). It will be important to understand the relative efficacy of the different Mef2 splice variants for their ability to regulate the expression level of miR‐134. However, validation of the interaction of Mef2 with its putative binding sites will require confirmation by additional approaches (e.g. chromatin immunoprecipitation or EMSA).

A lack of effect on *dmef2* transcript expression level following exposure to PTX is indicative that this factor does not, at least in *Drosophila*, form a primary link between neuron membrane depolarization and altered expression of *dpum*. However, we have been unable to source a usable dMef2 antibody and thus dMef2 protein levels were not determined. Our studies instead spotlight p300. p300 contains HAT activity and is also an accessory protein that interacts with transcription factors to function as either coactivator or repressor. For example, in *Drosophila*, p300 interacts with Dorsal, Mad and Cubitus interruptus, to coactivate *Toll*,* decapentaplegic* and *Hedgehog* signalling pathways respectively (Akimaru, Hou, & Ishii, 1997; Akimaru, Chen et al., 1997; Waltzer & Bienz, 1999). By contrast, T‐cell factor (TCF)‐mediated Wnt/Wingless signalling is repressed by p300 acetylation (Waltzer & Bienz, 1998). In mammals, p300 has been reported to bridge the complex of thyroid hormone receptor–retinoid X receptor–Mef2A to abrogate transactivation of α‐myosin heavy chain gene promoter activity when an inhibitor, adenovirus E1A for example, is recruited (De Luca et al., 2003). Here, we show p300 acts as a repressor of dMef2‐mediated transactivation of *dpum*. We further show that this effect is likely achieved through direct binding of p300 to dMef2. This result mirrors the reported physical interaction between a TAZ2 domain (within the cysteine–histidine‐rich region 3 (CH3)) of p300 and Mef2A/Mef2C (through MASD/Mef2 domains) in mammals (De Luca et al., 2003; He et al., 2011; Sartorelli et al., 1997). Lacking reliable anti‐dMef2 and anti‐p300 antibodies for *Drosophila*, we were not able to examine dMef2 and p300 protein–protein interaction by coimmunoprecipitation. However, a comparison of these two domains in human and *Drosophila* show 85% (TZA2 domains; Lin and Baines unpublished observation) and 86%–89% (MASD/Mef2 domains) amino acid identify (Lilly et al., 1994; Nguyen et al., 1994). These similarities support our yeast two‐hybrid data showing interaction between p300 and Mef2.

Our results are consistent with increasing neuronal depolarization negatively regulating the expression of p300 which, in turn, allows increased dMef2‐mediated transactivation of *dpum*. The expression of *p300* is known to be activity dependent and is similarly downregulated in pilocarpine (increased acetylcholine signalling) treated mouse hippocampus (Hansen, Sakamoto, Pelz, Impey, & Obrietan, 2014). The expression of *mef2* has also been reported to be regulated by increased synaptic excitation in mammals (Mao, Bonni, Xia, Nadal‐Vicens, & Greenberg, 1999), an observation we could not validate in *Drosophila*. We cannot rule out, however, that additional post‐transcriptional and/or post‐translational modifications, including alternative splicing and/or phosphorylation of *dmef2* might influence its activity. In this regard, a report of Mef2 activation by Ca^2+^‐activated dephosphorylation, via calcineurin is particularly attractive (Flavell et al., 2006). This is because Ca^2+^ entry across the neuronal membrane is widely regarded as an initial reporter of neuronal activity in homeostatic mechanisms (Cudmore & Turrigiano, 2004; Gunay & Prinz, 2010; O'Leary, van Rossum, & Wyllie, 2010). Alternative post‐transcriptional modifications of Mef2 activity have also been reported. For example, p38 mitogen‐activated protein kinase (p38‐MAPK) induced phosphorylation of Mef2C is critical for activation of Mef2 target genes (Han, Jiang, Li, Kravchenko, & Ulevitch, 1997; Mao et al., 1999).

The upregulation of *dpum* transcript expression in late stage 17 *Drosophila* embryos, due to increased synaptic excitation, was previously reported (Mee et al., 2004). Intriguingly, the analysis of transcript expression in the third instar CNS between wild type and wild type raised on food containing PTX revealed a significant reduction in *dpum* transcript expression (Lin et al., 2017). These paradoxical results might be a result of *dpum* autoregulation. The *dpum* transcript contains multiple PRE motifs in its 3'UTR region (Chen et al., 2008; Gerber, Luschnig, Krasnow, Brown, & Herschlag, 2006). Similarly, human *PUM1* and *PUM2* are also potential targets of PUM protein (Bohn et al., 2018). In this study, we used a *dpum* promoter to drive *firefly*‐luc expression, which lacks PRE motifs, and showed enhanced *dpum* promoter activity in third instar larvae raised on food containing PTX. This result validates that the *dpum* promoter is responsive to levels of synaptic activity and also provides additional evidence to suggest that Pum regulates its own expression through negative feedback.

**Figure 7:**
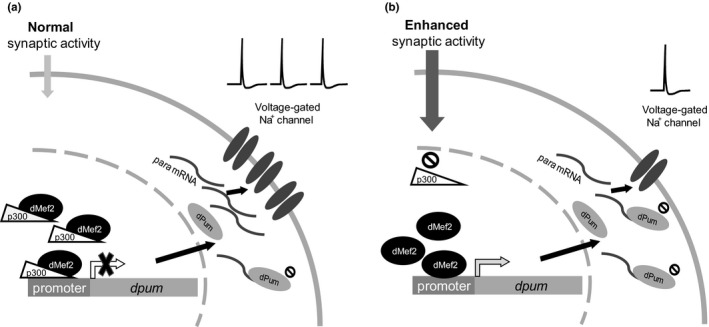
Transcription of *dpum* is coregulated by dMef2 and p300. (a) Under “normal” synaptic excitation, p300 is more abundant and binds to dMef2 to inhibit the transactivation of *dpum*. (b) Increasing exposure to synaptic excitation is sufficient to downregulate the expression of *p300* resulting in the release of dMef2 from inhibition. This facilitates transactivation of *dpum*. Increased dPum protein can, in turn, translationally repress *paralytic* mRNA (*para*;* voltage‐gated sodium channel*) to achieve a homeostatic reduction in action potential firing

In summary, we show that regulation of *dpum* expression is mediated by an interaction between dMef2 and p300, the latter being an activity‐dependent negative regulator. Under “normal” synaptic excitation, p300 is more abundant and binds to dMef2 to inhibit transactivation of *dpum*. Increased synaptic excitation reduces *p300* expression which, in turn, releases dMef2 from inhibition. The increase in dPum protein translationally represses *paralytic* (voltage‐gated sodium channel) mRNA to achieve a homeostatic reduction in action potential firing (a schematic mechanism is shown in Figure 7). It follows, therefore, that inhibition of p300 would be predicted to be anticonvulsant (mirroring increased dPum activity). Indeed, treatments (genetic or pharmacological) that elevate dPum activity are potently anticonvulsive in *Drosophila* seizure mutants (Lin et al., 2017). Moreover, seizures in flies, rodents and human are associated with decreased dPum or Pum activity respectively (Follwaczny et al., 2017; Lin et al., 2017; Siemen et al., 2011; Wu et al., 2015). Thus, while drug interventions that directly activate Pum may be difficult to achieve in the clinic, inhibition of p300 may represent a more achievable route to better control epilepsy.

## CONFLICT OF INTEREST

The authors have no competing interests to declare.

## AUTHORS’ CONTRIBUTION

WHL and RAB designed the study and wrote the paper. WHL performed the experiments and data analysis. RAB edited and approved the manuscript.

## Data Availability

All raw data are available upon request. Please contact RAB (Richard.Baines@manchester.ac.uk).
