# Supplementary material for: Myocyte enhancer factor‐2 and p300 interact to regulate the expression of homeostatic regulator Pumilio in Drosophila
Source: Eur J Neurosci. 2019 Feb 21;50(1):1727–40. doi: 10.1111/ejn.14357 (PMC6767705; doi:10.1111/ejn.14357)
Supplement: Supplementary file 1 [file EJN-50-1727-s001.pdf]

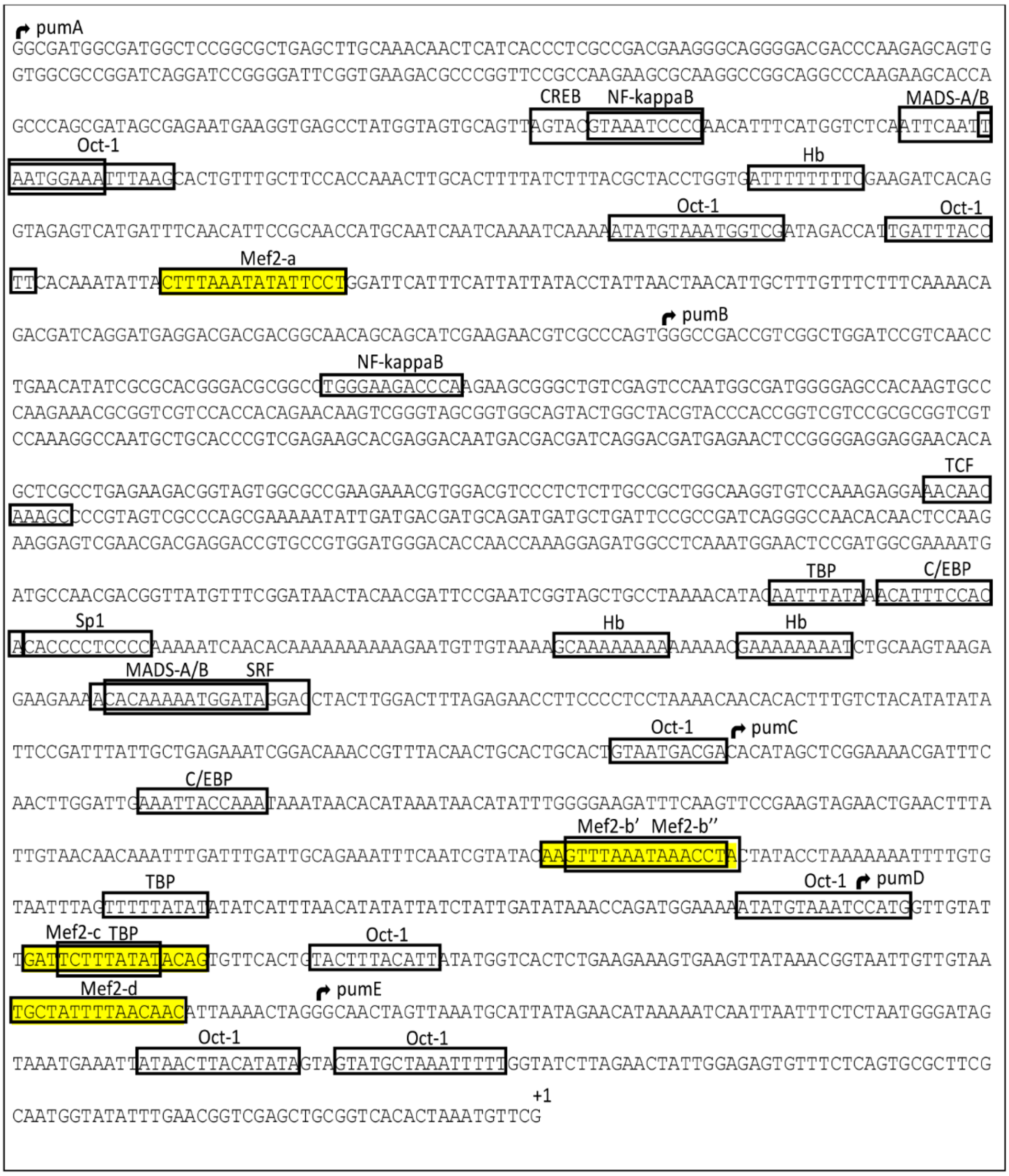

Supplementary Figure 1. Putative transcription factors identified in the *dpum* promoter

A 2-kb region upstream of the transcription start site of *dpum* was interrogated using transcription factor databases (e.g. TRANSFAC model, MAPPER) (21). In total 114 putative transcription factors within the region between -2000 and +1 (transcription initiation marked as +1) were identified. Putative transcriptional elements including, Sp1, TBP, C/EBP, Oct-1, Mef2, MADS-A/B, Hb, NF-kappaB, TCF, CREB and SRF are highlighted. Four Mef2 binding sites, Mef2-a, -b, -c and -d, are highlighted in yellow. Two predicted Mef2 binding motifs, Mef2-b’ and Mef2-b’’ overlap and, therefore, we count this as one site (Mef2-b). The initiation of each *dpum* promoter fragment, *pumA-E*, are indicated as black arrows.

Supplementary Table 1. Putative transcription factor-binding sites within the *drosophila pumilio* promoter region between -2000 and +1

Model	Factor	Strand	Start	End	Score	Eval
M00923	Adf-1	-	-1997	-1977	5.4	7.3
M00979	Pax-6	+	-1977	-1964	4.0	22.0
M00044	Sn	-	-1970	-1957	2.6	19.0
M00720	CAC-binding protein	+	-1941	-1933	2.5	20.0
M00448	Zic1	-	-1932	-1924	3.4	7.3
M00752	PDR3	-	-1897	-1890	1.5	24.0
M00247	PacC	+	-1871	-1855	2.6	16.0
M00806	NF-1	+	-1867	-1861	3.4	23.0
M00979	Pax-6	-	-1837	-1824	4.1	21.0
M00027	AbaA	-	-1821	-1806	6.0	6.4
M00616	AFP1	-	-1792	-1782	3.7	6.0
M00114	Tax/CREB	-	-1784	-1771	4.8	13.0
M00054	NF-kappaB	-	-1779	-1770	4.1	21.0
M00052	NF-kappaB (p65)	-	-1779	-1770	3.8	19.0
M00155	ARP-1 (COUP-TF2)	-	-1768	-1753	2.4	19.0
M00408	MADS-A	+	-1752	-1737	2.9	13.0
M00404	MADS-B	-	-1752	-1738	2.8	25.0
M00404	MADS-B	+	-1751	-1737	3.1	21.0
M00099	S8	+	-1749	-1743	4.4	12.0
M00135	Oct-1	+	-1745	-1731	3.4	20.0
M00930	Oct-1	-	-1744	-1734	4.5	13.0
M00094	BR-C Z4	+	-1744	-1732	2.3	17.0
M00935	NF-AT	+	-1742	-1734	4.2	19.0
M00149	SBF-1	-	-1739	-1726	3.0	11.0
M00062	IRF-1	-	-1711	-1700	4.4	14.0
M00355	PBF	-	-1707	-1698	3.4	21.0
M00725	HP1 site factor	-	-1686	-1675	4.0	6.4
M00022	Hb	-	-1680	-1671	3.0	25.0
M00821	Nrf-2	+	-1660	-1649	3.1	23.0
M00109	C/EBPbeta	-	-1650	-1637	4.4	13.0
M00104	CDP CR1	-	-1627	-1619	3.2	23.0
M00124	Pbx-1b	+	-1624	-1613	11.2	0.2
M00096	Pbx-1	+	-1624	-1616	4.2	4.8
M00689	unc-86	+	-1622	-1613	1.9	22.0

M00639	HNF6	+	-1618	-1607	4.3	16.0
M00062	IRF-1	+	-1617	-1606	3.3	24.0
M00242	PPARalpha:RXRalpha	+	-1612	-1593	5.6	7.4
M00161	Oct-1	-	-1608	-1595	4.0	13.0
M00135	Oct-1	+	-1607	-1593	5.3	10.0
M00464	POU3F2	+	-1607	-1598	4.0	12.0
M00795	Octamer	-	-1606	-1598	4.7	11.0
M00930	Oct-1	-	-1606	-1596	3.4	25.0
M00696	En	-	-1602	-1596	2.3	21.0
M00930	Oct-1	+	-1583	-1573	4.2	16.0
M00802	Pit-1	+	-1575	-1559	4.1	18.0
M00423	FOXJ2	-	-1571	-1558	4.1	4.9
M00094	BR-C Z4	-	-1565	-1553	2.6	13.0
M00094	BR-C Z4	+	-1562	-1550	2.5	14.0
M00006	MEF-2	+	-1561	-1546	3.7	17.0
M00699	ICSBP	-	-1546	-1535	3.6	16.0
M00747	IRF-1	+	-1542	-1536	3.3	17.0
M00621	C/EBPdelta	+	-1540	-1529	4.1	16.0
M00802	Pit-1	+	-1538	-1522	8.5	1.1
M00708	NIL	+	-1538	-1531	2.8	6.5
M00820	HAHB-4	-	-1536	-1528	4.4	18.0
M00081	Evi-1	-	-1533	-1519	3.3	13.0
M00696	En	+	-1525	-1519	2.2	25.0
M00274	STE11	+	-1513	-1497	7.0	3.2
M00923	Adf-1	-	-1472	-1452	6.3	4.1
M00975	RFX	-	-1466	-1458	2.6	14.0
M00391	UAY	-	-1424	-1409	4.0	7.2
M00644	LBP-1	+	-1422	-1416	1.1	13.0
M00774	NF-kappaB	+	-1377	-1366	5.0	12.0
M00043	dl	-	-1376	-1367	5.1	11.0
M00054	NF-kappaB	+	-1376	-1367	3.7	25.0
M00084	MZF1	+	-1343	-1331	3.4	18.0
M00929	MyoD	+	-1331	-1316	3.9	19.0
M00984	PEBP	+	-1305	-1291	3.8	19.0
M00273	R	-	-1304	-1286	3.3	23.0
M00731	Osf2	+	-1301	-1294	3.0	13.0
M00751	AML1	-	-1301	-1296	2.1	9.8
M00732	CAT8	+	-1254	-1245	3.0	23.0

M00411	HNF4alpha1	+	-1237	-1224	5.6	8.3
M00752	PDR3	-	-1168	-1161	1.5	24.0
M00261	Olf-1	+	-1151	-1132	3.7	20.0
M00644	LBP-1	+	-1095	-1089	1.1	13.0
M00474	FOXO1	-	-1076	-1064	4.5	17.0
M00269	XFD-3	+	-1076	-1063	3.9	9.1
M00275	Mat1-Mc	-	-1072	-1055	3.5	19.0
M00978	LEF1, TCF1	-	-1070	-1060	5.4	8.8
M00745	NIL	+	-1068	-1059	3.2	19.0
M00105	CDP CR3	+	-1047	-1033	5.8	7.3
M00120	dl	+	-1046	-1036	3.3	23.0
M00423	FOXJ2	-	-1042	-1029	3.5	8.0
M00937	TGA1a	+	-1035	-1014	3.5	15.0
M00777	STAT	-	-985	-978	4.7	15.0
M00238	Barbie Box	+	-938	-924	4.5	12.0
M00069	YY1	-	-937	-921	4.8	9.2
M00793	YY1	-	-931	-923	2.6	22.0
M00651	NF-muE1	-	-929	-921	2.5	21.0
M00130	FOXD3	-	-836	-826	4.8	14.0
M00471	TBP	-	-828	-821	5.0	12.0
M00802	Pit-1	+	-827	-811	4.6	13.0
M00268	XFD-2	+	-826	-813	4.1	6.7
M00267	XFD-1	+	-826	-813	3.4	12.0
M00094	BR-C Z4	+	-826	-814	3.1	7.5
M00129	HFH1 (FOXQ1)	-	-825	-815	6.0	6.1
M00472	FOXO4	+	-823	-813	4.3	19.0
M00465	POU6F1	-	-823	-813	3.7	20.0
M00109	C/EBPbeta	-	-819	-806	4.9	9.6
M00159	C/EBP	-	-819	-809	2.3	20.0
M00650	MTF-1	+	-816	-803	3.9	20.0
M00932	Sp1	-	-808	-798	4.4	18.0
M00931	Sp1	-	-807	-798	4.5	18.0
M00665	Sp3	-	-805	-792	3.8	8.7
M00649	MAZ	-	-805	-798	3.4	16.0
M00095	CDP	+	-799	-788	3.8	25.0
M00274	STE11	-	-786	-770	3.7	19.0
M00022	Hb	+	-762	-753	4.4	9.8
M00022	Hb	+	-746	-737	3.0	25.0

M00078	Evi-1	+	-727	-712	5.4	8.2
M00408	MADS-A	+	-718	-703	5.0	2.2
M00404	MADS-B	+	-717	-703	6.2	2.4
M00810	SRF	+	-716	-699	5.3	9.8
M00922	SRF	+	-715	-701	5.6	8.0
M00967	HNF4, COUP	-	-694	-687	3.4	15.0
M00658	PU.1	-	-678	-671	3.7	15.0
M00154	STRE	-	-676	-671	2.5	23.0
M00141	Lyf-1	-	-673	-665	3.9	8.0
M00456	FAC1	+	-665	-655	3.5	14.0
M00267	XFD-1	-	-657	-644	3.0	16.0
M00802	Pit-1	+	-650	-634	3.9	21.0
M00459	STAT5B (homodimer)	-	-631	-618	3.5	23.0
M00632	GATA-4	+	-623	-612	2.9	14.0
M00647	LXRalpha:RXRalpha	-	-593	-576	4.0	17.0
M00710	Zen	-	-588	-581	2.0	17.0
M00137	Oct-1	+	-587	-578	3.0	23.0
M00036	v-Jun	+	-586	-572	3.8	16.0
M00010	Opaque-2	-	-558	-543	5.0	5.2
M00091	BR-C Z1	+	-546	-529	3.3	16.0
M00274	STE11	-	-543	-527	3.8	19.0
M00159	C/EBP	-	-543	-533	3.0	11.0
M00681	WRKY	-	-540	-530	2.8	14.0
M00724	HNF3alpha	-	-537	-527	4.1	23.0
M00267	XFD-1	+	-536	-523	4.1	6.6
M00489	Nkx6-2	+	-535	-525	1.9	15.0
M00463	POU3F2	-	-533	-521	3.4	24.0
M00616	AFP1	+	-533	-523	3.2	9.1
M00456	FAC1	+	-530	-520	3.0	21.0
M00091	BR-C Z1	+	-524	-507	2.8	24.0
M00464	POU3F2	-	-519	-510	3.9	12.0
M00658	PU.1	+	-508	-501	3.3	21.0
M00657	PTF1-beta	+	-506	-493	3.3	17.0
M00453	IRF-7	-	-504	-489	5.1	11.0
M00747	IRF-1	-	-481	-475	3.0	22.0
M00149	SBF-1	-	-471	-458	3.3	8.6
M00622	C/EBPgamma	+	-450	-438	3.5	11.0
M00641	HSF	+	-446	-434	3.3	14.0

M00474	FOXO1	-	-432	-420	4.1	22.0
M00078	Evi-1	+	-427	-412	4.3	13.0
M00405	MEF-2	+	-423	-408	6.5	3.4
M00672	TEF	+	-423	-412	2.7	13.0
M00406	MEF-2	+	-421	-407	3.6	23.0
M00809	FOX factors	-	-418	-406	5.5	7.9
M00093	BR-C Z3	-	-384	-373	3.1	12.0
M00713	TBP	+	-376	-368	4.5	13.0
M00081	Evi-1	-	-376	-362	3.5	10.0
M00407	RSRFC4	+	-374	-360	3.2	23.0
M00026	RSRFC4	+	-374	-361	3.1	23.0
M00010	Opaque-2	+	-364	-349	3.1	16.0
M00710	Zen	+	-364	-357	1.6	25.0
M00478	Cdc5	+	-363	-352	2.6	16.0
M00464	POU3F2	-	-361	-352	4.0	12.0
M00078	Evi-1	-	-360	-345	6.5	4.4
M00080	Evi-1	-	-355	-345	5.9	6.8
M00465	POU6F1	-	-351	-341	3.3	25.0
M00238	Barbie Box	+	-340	-326	3.6	22.0
M00935	NF-AT	+	-328	-320	4.3	18.0
M00161	Oct-1	-	-322	-309	4.0	13.0
M00135	Oct-1	+	-321	-307	6.0	6.3
M00464	POU3F2	+	-321	-312	4.0	12.0
M00930	Oct-1	-	-320	-310	5.0	9.9
M00795	Octamer	-	-320	-312	4.7	11.0
M00639	HNF6	-	-303	-292	4.5	14.0
M00268	XFD-2	-	-299	-286	3.6	10.0
M00405	MEF-2	-	-298	-283	3.9	17.0
M00129	HFH1 (FOXQ1)	+	-297	-287	4.6	14.0
M00713	TBP	+	-295	-287	5.2	9.2
M00930	Oct-1	+	-273	-263	4.7	12.0
M00681	WRKY	+	-264	-254	2.8	14.0
M00691	ATF1	+	-253	-243	2.9	23.0
M00747	IRF-1	-	-244	-238	3.4	16.0
M00394	Msx-1	+	-229	-221	1.9	7.3
M00407	RSRFC4	-	-216	-202	3.9	17.0
M00941	MEF-2	+	-214	-203	5.8	5.8
M00149	SBF-1	-	-211	-198	2.6	16.0

M00710	Zen	+	-201	-194	1.8	21.0
M00157	RORalpha2	+	-195	-184	5.9	5.4
M00274	STE11	-	-169	-153	3.4	22.0
M00639	HNF6	+	-159	-148	6.5	4.1
M00124	Pbx-1b	+	-155	-144	6.3	4.9
M00096	Pbx-1	+	-155	-147	2.7	17.0
M00510	Lhx3a	-	-154	-145	3.9	24.0
M00099	S8	+	-153	-147	4.4	12.0
M00510	Lhx3a	+	-152	-143	6.1	5.6
M00125	MCM1	-	-146	-131	4.1	22.0
M00708	NIL	-	-140	-133	1.9	18.0
M00094	BR-C Z4	+	-133	-121	2.1	20.0
M00772	IRF	-	-130	-116	4.7	15.0
M00972	IRF	+	-130	-120	4.3	19.0
M00710	Zen	-	-130	-123	1.7	23.0
M00747	IRF-1	-	-128	-122	3.3	17.0
M00162	Oct-1	+	-118	-105	2.1	17.0
M00135	Oct-1	+	-101	-87	6.3	5.1
M00776	SREBP	+	-15	-7	3.4	20.0
M00749	SREBP-1	+	-13	-7	1.6	14.0

Supplementary Table 2. Putative transcription factor-binding sites within the mouse *pum2* promoter region between -2000 and +1

Model	Factor	Strand	Start	End	Score	Eval
M00057	COMP1	+	-1982	-1960	2.8	23.0
M00438	ARF	-	-1972	-1965	2.8	9.5
M00135	Oct-1	+	-1960	-1946	3.4	20.0
M00664	STE12	+	-1944	-1938	2.0	2.7
M00678	Tel-2	-	-1932	-1923	2.6	12.0
M00946	TGA1b	+	-1923	-1914	2.0	19.0
M00944	CPRF-3	-	-1923	-1916	1.9	24.0
M00946	TGA1b	-	-1923	-1914	2.0	19.0
M00788	EmBP-1b	+	-1922	-1916	3.0	6.6
M00472	FOXO4	-	-1883	-1873	4.2	21.0
M00021	Kr	-	-1859	-1850	1.8	21.0
M00042	SOX5	+	-1847	-1840	3.7	19.0
M00109	C/EBPbeta	+	-1845	-1832	3.4	25.0
M00621	C/EBPdelta	-	-1844	-1833	3.4	24.0
M00115	Tax/CREB	-	-1790	-1776	4.4	9.9
M00793	YY1	-	-1788	-1780	2.6	22.0
M00137	Oct-1	+	-1772	-1763	3.2	19.0
M00423	FOXJ2	+	-1772	-1759	3.3	9.7
M00267	XFD-1	-	-1769	-1756	2.8	19.0
M00269	XFD-3	-	-1769	-1756	6.0	1.8
M00094	BR-C Z4	-	-1768	-1756	2.6	12.0
M00809	FOX factors	+	-1768	-1756	5.3	8.7
M00962	AR	+	-1758	-1750	2.8	18.0
M00808	Pax	-	-1738	-1728	3.9	5.8
M00060	Sn	-	-1730	-1720	3.9	25.0
M00044	Sn	+	-1729	-1716	4.7	3.5
M00268	XFD-2	-	-1727	-1714	3.8	8.8
M00632	GATA-4	+	-1718	-1707	3.8	6.6
M00247	PacC	-	-1685	-1669	2.6	16.0
M00974	SMAD	+	-1677	-1667	4.4	12.0
M00438	ARF	-	-1667	-1660	2.8	9.0
M00457	STAT5A (homodimer)	-	-1658	-1645	4.5	17.0
M00808	Pax	+	-1652	-1642	4.8	2.7
M00723	GAGA factor	+	-1616	-1606	3.2	23.0
M00666	Sry-beta	+	-1601	-1593	1.8	22.0
M00064	PHO4	-	-1594	-1585	3.3	13.0

M00942	CPRF-1	+	-1593	-1584	2.5	7.5
M00440	CG1	+	-1593	-1583	4.4	2.2
M00942	CPRF-1	-	-1593	-1584	2.5	7.5
M00366	EmBP-1	-	-1593	-1584	4.6	13.0
M00034	p53	-	-1587	-1568	1.4	18.0
M00414	AREB6	+	-1571	-1560	6.5	3.1
M00277	Lmo2 complex	-	-1571	-1563	4.9	13.0
M00060	Sn	-	-1571	-1561	5.1	12.0
M00412	AREB6	+	-1570	-1560	3.8	18.0
M00413	AREB6	+	-1569	-1560	4.2	13.0
M00404	MADS-B	+	-1557	-1543	3.2	19.0
M00105	CDP CR3	+	-1554	-1540	5.2	10.0
M00463	POU3F2	+	-1549	-1538	5.0	11.0
M00802	Pit-1	+	-1549	-1533	5.0	11.0
M00162	Oct-1	+	-1546	-1533	1.7	25.0
M00639	HNF6	-	-1545	-1534	3.8	21.0
M00405	MEF-2	-	-1543	-1528	4.7	10.0
M00941	MEF-2	-	-1535	-1524	4.2	16.0
M00482	Pitx2	-	-1525	-1515	4.3	5.8
M00763	PPAR direct repeat 1	-	-1517	-1505	3.4	21.0
M00463	POU3F2	+	-1501	-1488	3.9	19.0
M00662	SGF-3	-	-1500	-1494	2.1	13.0
M00033	p300	+	-1476	-1466	2.4	20.0
M00162	Oct-1	-	-1471	-1458	2.8	8.5
M00464	POU3F2	+	-1470	-1461	3.9	12.0
M00407	RSRFC4	-	-1469	-1455	3.0	25.0
M00406	MEF-2	-	-1469	-1455	4.9	12.0
M00672	TEF	-	-1448	-1437	2.3	19.0
M00707	TFIIA	+	-1447	-1436	1.9	17.0
M00932	Sp1	-	-1432	-1422	4.0	24.0
M00931	Sp1	-	-1431	-1422	4.1	22.0
M00649	MAZ	-	-1429	-1422	3.4	16.0
M00154	STRE	-	-1426	-1421	2.5	23.0
M00404	MADS-B	-	-1411	-1397	2.9	25.0
M00639	HNF6	-	-1388	-1377	3.8	21.0
M00616	AFP1	+	-1386	-1376	3.4	7.6
M00026	RSRFC4	+	-1357	-1344	6.2	5.4
M00967	HNF4, COUP	-	-1344	-1337	3.4	15.0

M00046	GCR1	+	-1341	-1333	2.0	22.0
M00746	Elf-1	-	-1340	-1329	4.0	8.7
M00655	PEA3	+	-1340	-1334	2.7	15.0
M00274	STE11	+	-1305	-1289	6.8	3.6
M00094	BR-C Z4	-	-1304	-1292	3.9	3.5
M00802	Pit-1	+	-1303	-1287	3.9	20.0
M00436	IPF1	+	-1298	-1287	3.0	13.0
M00937	TGA1a	+	-1295	-1274	4.6	6.6
M00157	RORalpha2	-	-1292	-1281	4.1	16.0
M00156	RORalpha1	-	-1292	-1282	4.5	17.0
M00454	MRF-2	-	-1282	-1273	2.8	24.0
M00354	Dof3	+	-1272	-1252	4.1	20.0
M00267	XFD-1	-	-1249	-1236	2.9	18.0
M00809	FOX factors	+	-1248	-1236	5.3	8.9
M00094	BR-C Z4	-	-1248	-1236	3.0	8.9
M00791	HNF3	-	-1247	-1236	4.5	15.0
M00724	HNF3alpha	+	-1245	-1235	5.5	8.5
M00639	HNF6	-	-1242	-1231	3.7	22.0
M00791	HNF3	-	-1235	-1224	4.5	15.0
M00268	XFD-2	-	-1233	-1220	2.7	21.0
M00094	BR-C Z4	-	-1232	-1220	2.3	17.0
M00639	HNF6	-	-1230	-1219	4.1	18.0
M00724	HNF3alpha	+	-1229	-1219	5.7	7.4
M00062	IRF-1	-	-1219	-1208	6.8	3.5
M00110	Elf-1	+	-1217	-1204	1.3	24.0
M00405	MEF-2	-	-1214	-1199	4.4	12.0
M00734	CIZ	-	-1213	-1205	4.8	7.7
M00979	Pax-6	+	-1177	-1164	8.2	1.3
M00808	Pax	+	-1172	-1162	4.9	2.3
M00978	LEF1, TCF1	+	-1163	-1153	4.0	20.0
M00979	Pax-6	+	-1148	-1135	6.7	3.7
M00727	SF1	+	-1147	-1140	3.7	13.0
M00005	AP-4	-	-1128	-1111	2.8	20.0
M00983	MAF	-	-1118	-1111	3.1	13.0
M00480	LUN-1	+	-1113	-1099	2.0	24.0
M00480	LUN-1	-	-1113	-1099	16.6	0.0
M00666	Sry-beta	-	-1108	-1100	1.8	21.0
M00623	Crx	-	-1104	-1092	4.4	13.0

M00482	Pitx2	-	-1104	-1094	5.3	2.6
M00008	Sp1	-	-1083	-1074	2.2	22.0
M00079	Evi-1	+	-1074	-1065	5.2	11.0
M00011	Evi-1	+	-1073	-1065	4.0	13.0
M00133	Tst-1	-	-1066	-1054	2.1	11.0
M00622	C/EBPgamma	-	-1065	-1053	4.0	7.4
M00701	SMAD3	+	-1054	-1046	3.6	12.0
M00802	Pit-1	-	-1048	-1032	6.8	3.5
M00137	Oct-1	+	-1043	-1034	3.2	19.0
M00820	HAHB-4	+	-1042	-1034	5.6	8.2
M00480	LUN-1	-	-1031	-1016	4.2	8.6
M00985	Stra13	-	-998	-988	4.3	17.0
M00443	Opaque-2	-	-996	-985	2.9	25.0
M00274	STE11	-	-993	-977	4.1	16.0
M00978	LEF1, TCF1	-	-991	-981	3.9	21.0
M00745	NIL	+	-989	-980	2.9	24.0
M00480	LUN-1	+	-954	-938	5.7	4.3
M00105	CDP CR3	+	-953	-939	4.0	22.0
M00501	ANT	+	-953	-940	6.9	3.4
M00641	HSF	-	-949	-937	2.8	20.0
M00977	EBF	-	-947	-937	2.3	18.0
M00437	CHX10	-	-931	-921	5.0	12.0
M00478	Cdc5	+	-930	-919	2.2	22.0
M00510	Lhx3a	+	-929	-920	7.4	2.3
M00099	S8	-	-927	-921	4.2	14.0
M00099	S8	+	-926	-920	4.2	14.0
M00708	NIL	-	-926	-919	1.7	22.0
M00671	TCF-4	-	-923	-916	1.8	15.0
M00016	E74A	+	-901	-887	4.1	18.0
M00678	Tel-2	-	-898	-889	3.5	4.9
M00023	HOXA5 (Hox-1.3)	-	-896	-867	6.3	4.6
M00018	Ubx	-	-890	-882	5.3	10.0
M00489	Nkx6-2	+	-886	-876	1.5	22.0
M00510	Lhx3a	+	-886	-877	5.9	6.8
M00099	S8	-	-884	-878	4.2	14.0
M00437	CHX10	+	-883	-873	3.8	23.0
M00099	S8	+	-883	-877	4.3	14.0
M00016	E74A	+	-882	-868	5.5	8.3

M00655	PEA3	-	-878	-872	2.7	15.0
M00634	GCM	-	-866	-855	2.6	19.0
M00410	SOX9	-	-796	-787	5.5	8.9
M00728	ROX1	+	-795	-787	4.4	16.0
M00978	LEF1, TCF1	+	-794	-784	3.9	21.0
M00042	SOX5	-	-794	-787	3.7	18.0
M00079	Evi-1	+	-763	-754	4.6	15.0
M00790	HNF1	-	-746	-729	3.7	25.0
M00269	XFD-3	+	-738	-725	3.1	18.0
M00809	FOX factors	-	-738	-726	5.1	9.9
M00130	FOXD3	-	-737	-727	5.3	9.8
M00772	IRF	+	-707	-693	4.3	18.0
M00699	ICSBP	-	-705	-694	4.2	10.0
M00984	PEBP	-	-700	-686	3.5	23.0
M00711	Zta	+	-682	-670	4.2	10.0
M00459	STAT5B (homodimer)	+	-667	-654	3.5	23.0
M00457	STAT5A (homodimer)	+	-667	-654	5.9	6.4
M00746	Elf-1	+	-666	-655	3.7	12.0
M00007	Elk-1	+	-665	-651	2.3	14.0
M00531	NERF1a	+	-665	-648	4.9	6.7
M00108	NRF-2/GABP	+	-663	-654	2.2	25.0
M00046	GCR1	-	-662	-654	2.0	22.0
M00743	c-Ets-1 68	+	-662	-655	2.9	15.0
M00678	Tel-2	-	-662	-653	3.9	3.3
M00032	c-Ets-1(p54)	+	-662	-654	3.7	15.0
M00771	Ets	-	-662	-655	5.7	7.5
M00655	PEA3	-	-661	-655	2.7	15.0
M00515	PPARgamma:RXRalpha, PPARgamma	-	-658	-636	2.8	12.0
M00447	AR	-	-629	-615	2.8	25.0
M00753	RCS1	-	-627	-615	3.9	12.0
M00408	MADS-A	-	-622	-607	2.7	16.0
M00081	Evi-1	-	-617	-603	3.3	12.0
M00022	Hb	-	-612	-603	4.0	13.0
M00934	Zeste	+	-589	-580	6.8	3.6
M00444	VDR	+	-583	-569	2.9	23.0
M00154	STRE	+	-576	-571	2.5	23.0
M00941	MEF-2	+	-551	-540	3.9	18.0
M00016	E74A	+	-514	-500	6.8	3.6

M00108	NRF-2/GABP	+	-512	-503	3.0	13.0
M00743	c-Ets-1 68	+	-511	-504	3.3	11.0
M00032	c-Ets-1(p54)	+	-511	-503	4.0	12.0
M00678	Tel-2	-	-511	-502	4.2	2.3
M00025	Elk-1	+	-511	-502	5.2	11.0
M00655	PEA3	-	-510	-504	2.4	21.0
M00084	MZF1	+	-508	-496	4.6	7.5
M00404	MADS-B	-	-490	-476	3.3	18.0
M00633	GBF	+	-483	-475	2.5	7.7
M00749	SREBP-1	-	-481	-475	1.7	13.0
M00670	NIL	+	-450	-443	1.8	9.2
M00141	Lyf-1	+	-444	-436	2.6	21.0
M00275	Mat1-Mc	-	-441	-424	3.6	18.0
M00162	Oct-1	+	-436	-423	1.8	24.0
M00728	ROX1	-	-436	-428	4.7	13.0
M00042	SOX5	+	-436	-429	3.7	18.0
M00410	SOX9	+	-436	-427	5.5	8.8
M00708	NIL	-	-434	-427	1.9	18.0
M00632	GATA-4	+	-429	-418	3.7	7.3
M00531	NERF1a	+	-422	-405	3.4	19
M00771	Ets	-	-419	-412	4	24
M00681	WRKY	-	-409	-399	2.3	20
M00744	POU1F1	+	-406	-397	6.2	4.5
M00463	POU3F2	+	-406	-393	6.5	4.4
M00463	POU3F2	-	-406	-393	8.8	0.88
M00662	SGF-3	-	-405	-399	1.6	21
M00274	STE11	-	-394	-378	3.5	21
M00670	NIL	+	-376	-369	0.9	23
M00115	Tax/CREB	-	-376	-362	4.8	7.5
M00479	Alfin1	-	-367	-357	2.5	23
M00751	AML1	-	-363	-358	2.1	9.8
M00237	AhR:Arnt	+	-358	-340	5.9	6.7
M00448	Zic1	-	-347	-339	2.2	22
M00650	MTF-1	-	-342	-329	4.2	17
M00723	GAGA factor	+	-333	-323	3.4	19
M00158	COUP-TF, HNF-4	-	-329	-316	3.8	22
M00765	COUP direct repeat 1	-	-328	-316	3.3	25
M00763	PPAR direct repeat 1	-	-328	-316	3.4	21

M00629	Eve	-	-310	-301	1.6	25
M00720	CAC-binding protein	-	-306	-298	2.7	17
M00777	STAT	-	-295	-288	5.2	10
M00249	CHOP:C/EBPalpha	-	-277	-267	4	22
M00069	YY1	-	-271	-255	5.3	6.6
M00733	SMAD4	-	-263	-249	3.2	21
M00651	NF-muE1	-	-263	-255	4.2	5.5
M00652	NRF-1	-	-225	-216	6.8	2.4
M00778	AhR	-	-224	-215	3.7	23
M00652	NRF-1	+	-223	-214	3.5	19
M00235	AhR:Arnt	-	-222	-215	3.9	18
M00650	MTF-1	+	-197	-184	5.7	7.5
M00716	ZF5	+	-187	-180	3.1	11
M00433	Hmx3 (Nkx5-1)	-	-173	-164	3.1	21
M00247	PacC	+	-164	-148	2.5	19
M00644	LBP-1	+	-149	-143	1.1	13
M00632	GATA-4	-	-134	-123	3.2	11
M00800	AP-2	-	-115	-100	3.4	22
M00650	MTF-1	+	-112	-99	6.8	3.6
M00333	ZF5	-	-111	-100	2.6	15
M00716	ZF5	+	-109	-102	3.1	11
M00721	CACCC-binding factor	+	-104	-89	4.7	5.9
M00915	AP-2	-	-103	-91	4	18
M00800	AP-2	+	-102	-87	3.7	19
M00933	Sp1	-	-98	-89	5.1	11
M00807	Egr	+	-98	-88	3	15
M00938	E2F-1	+	-97	-82	3.3	19
M00982	KROX	-	-97	-84	3.7	24
M00932	Sp1	+	-97	-87	4.7	15
M00444	VDR	+	-92	-78	3.2	18
M00649	MAZ	+	-91	-84	3.4	16
M00706	TFII-I	+	-89	-81	1.9	12
M00154	STRE	+	-83	-78	2.5	23
M00649	MAZ	+	-82	-75	3.4	16
M00982	KROX	-	-80	-67	3.9	21
M00923	Adf-1	-	-76	-56	8.3	1.2
M00695	ETF	+	-71	-65	2.8	21
M00069	YY1	+	-61	-45	3.3	24

M00793	YY1	+	-59	-51	3.9	8.3
M00808	Pax	-	-48	-38	2.2	25
M00721	CACCC-binding factor	-	-35	-20	2.7	25

Supplementary Table 3. Putative transcription factor-binding sites within the human *pum2* promoter region between -2000 and +1

Model	Factor	Strand	Start	End	Score	Eval
M00506	LIM1	+	-1996	-1985	2.5	14.0
M00753	RCS1	+	-1994	-1982	3.4	17.0
M00407	RSRFC4	-	-1978	-1964	4.3	15.0
M00408	MADS-A	-	-1977	-1962	2.9	14.0
M00404	MADS-B	-	-1977	-1963	5.9	3.0
M00026	RSRFC4	+	-1975	-1962	4.7	12.0
M00666	Sry-beta	-	-1963	-1955	1.8	22.0
M00054	NF-kappaB	+	-1957	-1948	3.9	22.0
M00143	Pax-5	-	-1937	-1910	3.8	17.0
M00683	XBP1	-	-1929	-1924	2.8	14.0
M00518	PPARalpha:RXRalpha	-	-1929	-1915	5.6	8.3
M00155	ARP-1 (COUP-TF2)	+	-1927	-1912	3.9	6.7
M00963	T3R	+	-1921	-1913	3.5	17.0
M00647	LXRalpha:RXRalpha	-	-1919	-1902	5.8	5.6
M00766	LXR direct repeat 4	+	-1919	-1904	9.2	0.6
M00733	SMAD4	-	-1912	-1898	3.4	18.0
M00932	Sp1	-	-1909	-1899	4.1	22.0
M00807	Egr	-	-1908	-1898	2.5	23.0
M00931	Sp1	-	-1908	-1899	4.4	19.0
M00933	Sp1	+	-1907	-1898	4.5	17.0
M00982	KROX	+	-1906	-1893	4.1	19.0
M00700	ROM	+	-1902	-1894	3.2	6.4
M00506	LIM1	+	-1901	-1890	2.3	17.0
M00141	Lyf-1	-	-1892	-1884	4.9	3.3
M00480	LUN-1	-	-1890	-1875	19.7	0.0
M00666	Sry-beta	-	-1884	-1876	1.8	21.0
M00482	Pitx2	-	-1880	-1870	4.8	4.1
M00717	Pax-8	+	-1868	-1861	1.5	20.0
M00725	HP1 site factor	-	-1838	-1827	3.1	13.0
M00006	MEF-2	+	-1837	-1822	3.9	15.0
M00406	MEF-2	+	-1836	-1822	6.3	5.1
M00753	RCS1	+	-1831	-1819	3.2	20.0
M00018	Ubx	-	-1803	-1795	4.2	20.0
M00478	Cdc5	+	-1766	-1755	3.3	7.7
M00465	POU6F1	-	-1764	-1754	3.8	18.0
M00464	POU3F2	-	-1764	-1755	3.9	13.0

M00689	unc-86	-	-1756	-1747	2.2	16.0
M00725	HP1 site factor	+	-1750	-1739	3.7	8.3
M00005	AP-4	-	-1748	-1731	2.6	24.0
M00626	RFX1 (EF-C)	-	-1746	-1733	2.4	18.0
M00485	Nkx2-2	-	-1710	-1701	4.2	18.0
M00105	CDP CR3	-	-1668	-1654	4.1	20.0
M00935	NF-AT	+	-1655	-1647	4.5	16.0
M00749	SREBP-1	+	-1604	-1598	2.0	9.5
M00154	STRE	+	-1577	-1572	2.5	23.0
M00649	MAZ	+	-1576	-1569	3.4	16.0
M00960	PR, GR	+	-1571	-1562	4.6	11.0
M00930	Oct-1	+	-1541	-1531	3.5	24.0
M00977	EBF	+	-1537	-1527	2.0	23.0
M00769	AML	+	-1533	-1522	3.9	21.0
M00751	AML1	+	-1531	-1526	2.1	9.8
M00722	core-binding factor	+	-1531	-1524	3.2	12.0
M00501	ANT	-	-1511	-1498	7.7	1.9
M00975	RFX	-	-1499	-1491	2.6	14.0
M00093	BR-C Z3	-	-1489	-1478	2.9	15.0
M00011	Evi-1	-	-1489	-1481	3.7	16.0
M00268	XFD-2	-	-1487	-1474	3.3	12.0
M00472	FOXO4	-	-1487	-1477	4.0	24.0
M00269	XFD-3	-	-1487	-1474	5.3	3.2
M00474	FOXO1	+	-1486	-1474	9.0	0.8
M00619	Alx-4	-	-1482	-1470	4.0	7.3
M00977	EBF	-	-1476	-1466	2.9	10.0
M00154	STRE	+	-1472	-1467	2.5	23.0
M00649	MAZ	+	-1471	-1464	3.4	16.0
M00355	PBF	-	-1454	-1445	3.4	22.0
M00275	Mat1-Mc	+	-1423	-1406	3.3	22.0
M00059	YY1	-	-1403	-1392	2.5	21.0
M00487	mtTFA	-	-1395	-1387	2.9	16.0
M00622	C/EBPgamma	+	-1381	-1369	4.0	7.3
M00045	E4BP4	-	-1379	-1369	4.3	17.0
M00616	AFP1	-	-1350	-1340	2.6	16.0
M00089	Athb-1	+	-1346	-1337	6.4	4.8
M00775	NF-Y	-	-1338	-1328	6.1	5.9
M00094	BR-C Z4	-	-1326	-1314	2.3	16.0

M00423	FOXJ2	-	-1317	-1304	2.2	25.0
M00626	RFX1 (EF-C)	-	-1306	-1293	2.2	22.0
M00053	c-Rel	-	-1298	-1289	3.5	19.0
M00034	p53	+	-1298	-1279	5.6	4.0
M00034	p53	-	-1298	-1279	5.0	5.0
M00011	Evi-1	-	-1286	-1278	5.1	7.2
M00161	Oct-1	-	-1276	-1263	4.6	8.7
M00795	Octamer	-	-1274	-1266	3.4	25.0
M00020	Ftz	+	-1267	-1256	3.2	25.0
M00708	NIL	+	-1265	-1258	2.2	13.0
M00806	NF-1	+	-1240	-1234	3.4	23.0
M00261	Olf-1	-	-1220	-1201	4.6	11.0
M00977	EBF	-	-1214	-1204	2.6	14.0
M00749	SREBP-1	-	-1191	-1185	1.7	13.0
M00133	Tst-1	+	-1184	-1172	1.6	19.0
M00267	XFD-1	-	-1149	-1136	3.6	10.0
M00135	Oct-1	-	-1149	-1135	3.9	17.0
M00672	TEF	+	-1146	-1135	2.1	22.0
M00930	Oct-1	+	-1146	-1136	4.0	18.0
M00724	HNF3alpha	+	-1145	-1135	4.3	19.0
M00795	Octamer	+	-1144	-1136	4.0	17.0
M00010	Opaque-2	+	-1119	-1104	5.0	5.2
M00405	MEF-2	-	-1108	-1093	4.3	14.0
M00405	MEF-2	-	-1091	-1076	4.2	15.0
M00696	En	+	-1089	-1083	3.0	12.0
M00640	HOXA4	-	-1089	-1082	2.1	8.8
M00099	S8	+	-1088	-1082	3.8	18.0
M00268	XFD-2	+	-1086	-1073	2.5	24.0
M00708	NIL	-	-1082	-1075	2.2	13.0
M00501	ANT	-	-1005	-992	5.4	9.6
M00444	VDR	+	-984	-970	3.1	19.0
M00721	CACCC-binding factor	+	-960	-946	2.8	24.0
M00720	CAC-binding protein	+	-955	-947	2.5	20.0
M00931	Sp1	+	-954	-945	4.5	17.0
M00932	Sp1	+	-954	-944	4.9	13.0
M00479	Alfin1	+	-953	-943	4.0	6.5
M00684	XPF-1	+	-929	-920	1.3	17.0
M00158	COUP-TF, HNF-4	-	-889	-876	3.7	22.0

M00161	Oct-1	+	-843	-830	3.2	22.0
M00269	XFD-3	-	-837	-824	2.7	24.0
M00268	XFD-2	-	-837	-824	3.2	14.0
M00463	POU3F2	-	-832	-820	3.7	22.0
M00408	MADS-A	-	-826	-811	2.2	25.0
M00744	POU1F1	+	-825	-816	3.5	24.0
M00463	POU3F2	+	-825	-812	6.1	5.8
M00162	Oct-1	+	-822	-809	3.0	7.4
M00639	HNF6	-	-819	-808	3.9	20.0
M00105	CDP CR3	+	-803	-789	3.8	23.0
M00006	MEF-2	+	-790	-775	3.3	22.0
M00129	HFH1 (FOXQ1)	-	-784	-774	4.8	13.0
M00438	ARF	+	-774	-767	2.2	17.0
M00484	Ncx	+	-746	-739	1.9	17.0
M00622	C/EBPgamma	+	-733	-721	4.3	6.0
M00772	IRF	-	-726	-712	6.1	5.6
M00699	ICSBP	+	-725	-714	5.4	4.5
M00162	Oct-1	-	-725	-712	2.5	12.0
M00161	Oct-1	-	-725	-712	6.2	2.9
M00747	IRF-1	-	-724	-718	3.3	17.0
M00795	Octamer	-	-723	-715	3.6	22.0
M00930	Oct-1	-	-723	-713	5.8	6.1
M00154	STRE	+	-713	-708	2.3	25.0
M00734	CIZ	+	-699	-691	3.9	14.0
M00059	YY1	-	-684	-673	2.3	25.0
M00036	v-Jun	+	-675	-661	3.7	18.0
M00916	CREB	+	-674	-661	4.8	14.0
M00691	ATF1	-	-672	-662	3.8	12.0
M00917	CREB	-	-672	-662	4.1	23.0
M00775	NF-Y	+	-658	-648	5.1	11.0
M00022	Hb	-	-641	-632	3.3	20.0
M00915	AP-2	+	-632	-620	7.3	2.4
M00800	AP-2	+	-630	-615	3.9	16.0
M00158	COUP-TF, HNF-4	-	-613	-600	4.4	15.0
M00985	Stra13	-	-605	-595	7.0	3.0
M00440	CG1	-	-604	-594	2.6	11.0
M00796	USF	-	-604	-593	6.4	4.3
M00942	CPRF-1	+	-603	-594	1.3	23.0

M00942	CPRF-1	-	-603	-594	1.3	23.0
M00946	TGA1b	+	-603	-594	2.1	17.0
M00946	TGA1b	-	-603	-594	2.1	17.0
M00440	CG1	+	-603	-593	2.8	8.9
M00944	CPRF-3	-	-603	-596	2.5	14.0
M00945	CPRF-2	-	-603	-596	3.1	21.0
M00366	EmBP-1	-	-603	-594	4.2	17.0
M00788	EmBP-1b	+	-602	-596	3.4	3.8
M00985	Stra13	+	-602	-592	5.2	11.0
M00727	SF1	+	-582	-575	3.7	13.0
M00710	Zen	-	-553	-546	1.6	25.0
M00463	POU3F2	-	-549	-536	3.4	25.0
M00091	BR-C Z1	+	-507	-490	2.9	21.0
M00274	STE11	-	-504	-488	3.6	20.0
M00275	Mat1-Mc	-	-504	-487	5.8	4.0
M00724	HNF3alpha	-	-502	-492	4.8	14.0
M00105	CDP CR3	+	-499	-485	3.6	25.0
M00267	XFD-1	+	-497	-484	3.1	15.0
M00488	DREF	-	-496	-483	3.4	22.0
M00941	MEF-2	-	-494	-483	3.5	24.0
M00354	Dof3	-	-487	-467	4.1	19.0
M00441	GBF	+	-483	-472	4.9	8.6
M00440	CG1	+	-482	-472	2.3	14.0
M00942	CPRF-1	+	-482	-473	3.7	2.4
M00366	EmBP-1	+	-482	-473	4.5	14.0
M00942	CPRF-1	-	-482	-473	3.7	2.4
M00366	EmBP-1	-	-482	-473	7.1	2.9
M00788	EmBP-1b	+	-481	-475	3.4	4.2
M00943	TAF-1	+	-480	-473	2.0	21.0
M00944	CPRF-3	+	-480	-473	2.1	20.0
M00945	CPRF-2	+	-480	-473	3.1	21.0
M00972	IRF	+	-452	-442	4.1	22.0
M00747	IRF-1	+	-442	-436	3.4	16.0
M00116	C/EBPalpha	-	-430	-420	3.3	19.0
M00159	C/EBP	-	-429	-419	2.1	25.0
M00135	Oct-1	-	-414	-400	4.4	14.0
M00223	STATx	+	-408	-400	3.7	21.0
M00027	AbaA	-	-398	-383	4.7	15.0

M00777	STAT	-	-396	-389	4.0	23.0
M00701	SMAD3	-	-365	-357	3.5	13.0
M00733	SMAD4	+	-364	-350	3.3	19.0
M00247	PacC	+	-360	-344	3.4	8.1
M00804	E2A	-	-349	-337	3.5	17.0
M00031	MATalpha2	+	-342	-334	2.6	7.8
M00974	SMAD	-	-254	-244	4.3	13.0
M00480	LUN-1	+	-238	-223	3.6	12.0
M00967	HNF4, COUP	+	-234	-227	3.7	12.0
M00683	XBP1	+	-220	-215	2.8	14.0
M00482	Pitx2	+	-204	-194	3.0	16.0
M00059	YY1	+	-191	-180	2.8	15.0
M00649	MAZ	+	-181	-174	3.1	20.0
M00931	Sp1	+	-181	-172	3.9	25.0
M00639	HNF6	-	-169	-158	4.5	14.0
M00011	Evi-1	-	-161	-153	6.4	3.3
M00751	AML1	-	-148	-143	2.1	10.0
M00749	SREBP-1	+	-146	-140	1.6	14.0
M00405	MEF-2	+	-134	-119	4	16
M00713	TBP	-	-130	-122	5.2	9.2
M00407	RSRFC4	-	-117	-103	2.9	25
M00026	RSRFC4	+	-114	-101	5.6	8.3
M00154	STRE	+	-95	-90	2.5	23
M00749	SREBP-1	+	-64	-58	1.7	13
M00622	C/EBPgamma	-	-26	-14	2.6	23
M00707	TFIIA	+	-24	-13	2.6	8.3
