# Supplementary material for: Myocyte enhancer factor‐2 and p300 interact to regulate the expression of homeostatic regulator Pumilio in Drosophila
Source: Eur J Neurosci. 2019 Feb 21;50(1):1727–40. doi: 10.1111/ejn.14357 (PMC6767705; doi:10.1111/ejn.14357)
Supplement: Supplementary file 2 [file EJN-50-1727-s002.docx]

| Myocyte enhancer factor-2 and p300 interact to regulate expression of the homeostatic regulator Pumilio in Drosophila | |
| --- | --- |
|  | Baines, Richard ; Lin, Wei-Hsiang |

_____________________________________________________________________________________

| Review timeline: | Submission date: 29/10/18  Editorial Decision: Minor Revision  Revision Received: 06/12/18  Editorial Decision: Minor Revision  Revision Received: 03/01/19  Accepted: 04/01/19 |  |
| --- | --- | --- |
|  |  |  |

Editor: Giovanni C. Galizia

Reviewer 1: Paolo Macchi

Reviewer 2: Fedor Karginov

_____________________________________________________________________________________

1st Editorial Decision	22/11/18

_____________________________________________________________________________________

Myocyte enhancer factor-2 and p300 interact to regulate expression of the homeostatic regulator Pumilio in Drosophila 

Dear Dr. Baines, 

Your manuscript has been reviewed by two external reviewers as well as by the Section Editor,  Dr. C. Giovanni Galizia, and ourselves. 

As you can see below, the reviewers are very complimentary about the study but you will also notice that there are a few minor revisions that need to be addressed in order for it to be suitable for publication in EJN. In particular, one reviewer notes some over-interpretations to be down-toned. The other reviewer would like to see independent evidence for the putative binding sites within dpum promoter. Please make sure to address each of the  reviewers' comments in your revised version. 

We also noted the following EJN style points that will need to be addressed in the revised version. 
- Please include 'funding' in the acknowledgments. 
- Include a data statement, a list of abbreviations and an author contributions statement. 
- In accordance with EJN guidelines please replace bar charts with more informative scatter plots or similar. 
- Please supply a graphic abstract and text (see below). 
- The figures need to be uploaded as separate files but the legends should be in the main text. 
- Remove numbers from the reference list. 

When revising the manuscript, please embolden or underline major changes to the text so they are easily identifiable and please don't leave 'track change' formatting marks in your paper.  Please ensure that you provide a text and a figure file for the Graphical Abstract (as detailed in the instructions below).  When carrying out your revisions please refer to the checklist below and visit the EJN author guidelines at www.ejneuroscience.org 

When finalized, please upload your complete revised manuscript onto the website, as a Word file (.doc, or .docx).  Please also ensure that a complete set of tables and figures is included as separate files, even if these have not changed from the originals.  At this stage it is necessary to provide high resolution figures.  Please see important instructions below. 

Please go into https://mc.manuscriptcentral.com/ejn - Author Centre - manuscripts with decisions where you will find a 'create a revision' link under 'actions'. We ask that you please indicate the way in which you have responded to the points raised by the Editors and Reviewers in a letter. Please upload this response letter as a separate Word (.doc or PDF) file using the file designation "Authors' Response to Reviewers" when uploading your manuscript files.  Please DO NOT submit your revised manuscript as a new one.  Also, please note that only the  Author who submitted the original version of the manuscript should submit a revised version. 

If you are able to respond fully to the points raised, we would be pleased to receive a revision of your paper within 30 days. 

Thank you for submitting your work to EJN. 

Best wishes, 

Paul Bolam & John Foxe 
co-Editors in Chief, EJN 


Reviews: 

Reviewer: 1 

Comments to the Author 
Pumilio (Pum) is an RNA-binding protein that is involved in the translation control of mRNAs containing specific sequences known as pumilio response element, usually located within their 3’UTR. Baines’s group has contributed to characterize Pum in Drosophila (dpum) showing that dpum can also play a role in neuronal firing-rate homeostastis. 
Several studies highlighted the biological function of Pum in vertebrates, but very little is known about how Pum is regulated at transcriptional level. In fact, although the levels of Pum in nerve cells are tightly regulated (e.g. by synaptic activity), its promoter has been poorly studied. 

In this paper, Lin and Baines characterize the promoter region of Drosophila Pumilio. 2 kb region upstream of dpum coding region was amplified and more than 100 putative binding motifs for transcription factors were identify by database analysis. Among these motifs, Mef2 binding elements were studied in a more detail. By using a luciferase reporter assay to monitor the promoter activity and transgenic flies, the authors show that Mef2 promotes the transcription by binding to four putative Mef2 binding sites within dpum promoter. Moreover, the authors tested 12 splice variants of Mef2 demonstrating that they were able to activate transcription. 
It is known that Pum levels increase upon synaptic excitation with a still-unknown mechanism. Lin and Baines nicely show that, although Mef2 levels are unaffected by stimulation with pictrotoxin, the mRNA encoding for transcription regulator p300 is downregulated. Interestingly, by using the two-hybrid assay, the authors demonstrate that p300 interacts with Mef2 and compete with its binding with dpum promoter leading to a reduction of dpum transcription. Thus, synaptic excitation regulates dpum transcription via a mutual interaction between Mef2 and p300. 

The paper is well written, the data are nicely presented and the figure legends are clear and exhaustive. The results are very interesting and they contribute to define the molecular mechanisms involved in dpum regulation upon synaptic stimulation and to identify molecular factors involved in regulating dpum transcription. Overall, I think the manuscript is an excellent contribution and appropriate for publication in European Journal of Neuroscience. 
There are only a few minor points of improvement that the authors should address or discuss. 

1- The interaction of Mef2 with its putative binding sites within dpum promoter should be confirmed by additional approaches (e.g. chromatin immunoprecipitation or by EMSA). Moreover, I am aware that good antibodies are missing, but co-IPs can be performed by over expressing tagged p300 and Mef2 either in the presence or in the absence of DNAse to confirm their direct protein-protein interaction. 

2- If p300 and Mef2 can be expressed in bacteria and then purified, the above-mentioned EMSA performed in the presence of increasing amount of p300 might validate the direct inhibitory effect of p300 on MEF2's DNA binding activity.  Moreover, with this approach it might possible to assess whether the Mef2 isoform containing the exon 10 – the isoform that shows the most robust transactivation activity - binds DNA with a more affinity. 

3-Page 18: please modify the “Supplementary data are available at NAR online.” 

Reviewer: 2 

Comments to the Author 
Lin and Baines investigate the transcriptional regulation of the drosophila Pum gene, demonstrating control by dMef2 and p300 in S2R+ cells, and place the regulation in the context of activity-dependent changes in the nervous system. The promoter region of dPum is mapped by luciferase reporter assays, and dMef2 sites are identified by bioinformatic analysis, with its effects on the reporters confirmed by knockdown and overexpression. Furthermore, reporter activity of a 3’ UTR Pum response element is affected by dMef2 expression, indicating regulation of endogenous dPum activity. dMef2 isoforms containing alternatively spliced exon 10 are found to induce higher transcription. To understand neuronal activity-dependent changes in dPum expression, transcript levels in control and PTX-fed larvae were measured; dmef2 does not increase, but its potential co-regulator p300 decreases (although the effect is not large). Overexpression of p300 reduces dPum promoter reporter activity, and abolishes the activation by dMef2 overexpression, while p300 knockdown induces reporter activity. A direct interaction between p300 and dMef2 is shown by yeast two-hybrid. Importantly, in vivo regulation of the pum promoter by dMef2 and p300 is demonstrated with luc transgene reporters. These findings are interesting due to the known involvement of Pum in neuronal development and homeostasis, and provide important clues to the regulatory networks that connect neuronal excitation with cellular responses, such as synaptic growth. The manuscript is well-written and experiments are generally well-designed and carried out, raising only minor concerns. There are a few concerns with interpretation of results, but I believe the manuscript would be of interest to the audience of the journal, and recommend publication after addressing the following points: 

- A trivial, but helpful bioinformatics control: in similar-sized promoter regions of a random selection of genes, how many dMef2 elements are seen on average, and what is the standard deviation? 
- Line 263 – In this section, it is somewhat of a leap to conclude that dPum levels are what is affected (although likely), since the assay is indirect. Since other post-translational ways of activating Pum are possible, it would be more correct to say that dPum activity is increased. It may be relevant to mention that it is the activity of endogenous Pum that is being affected. 
- Line 697 – “dMef2 binding elements are required for dpum promoter transactivation and increased dPum activity”. This is an overstatement, since the binding elements themselves were not tested by mutagenesis, and large sections of the promoter region were deleted instead. Whether dMef2 binding elements are required for increased dPum activity was not really tested at all. 
- Neuronal activity-dependent changes in dmef2 transcript levels are measured, but protein levels and phosphorylation status (which is important in other model systems) are not, so it is a stretch to conclude that dMef2 levels are not impacted by synaptic excitation in the results section. The possibilities of post-transcriptional control and effects of phosphorylation should be mentioned upfront. 
- Connections to the human regulatory model, where Mef2 induces miR-134 expression, thus repressing Pum2, could be discussed in further detail. Is there a drosophila homolog of miR-134, is there experimental evidence of dMef2 – miR-134 regulation? Are there dMef2 sites in the miR-134 promoter? 

------------------------

____________________________________________________________________________________

Authors’ Response

_____________________________________________________________________________________

Dear Editor 

We are submitting our revision for the above manuscript (EJN-2018-10-25978). 

The comments from both reviewers are as follows: 

Reviewer: 1 

This reviewer states ‘There are only a few minor points of improvement that the authors should address or discuss.’ 

1. The interaction of Mef2 with its putative binding sites within dpum promoter should be confirmed by additional approaches (e.g. chromatin immunoprecipitation or by EMSA). Moreover, I am aware that good antibodies are missing, but co-IPs can be performed by over expressing tagged p300 and Mef2 either in the presence or in the absence of DNAse to confirm their direct protein-protein interaction. 

Although, we have not performed additional experiments to further show the specificity of Mef2 binding to the dpum promoter, the series of dpum promoter deletions clearly show that the Mef2 binding site is required for promoter transactivation. Moreover, a genome-wide tiling array analysis similarly identified dpum as a target of dMef2 (Sivachenko et al., 2013). In the same way, we analysed human and mouse Pum2 promoter regions and identified Mef2 elements. More importantly, ChIP-X enrichment analysis shows Mef2A associates with the Pum2 promoter region (Lachmann et al., 2010; Rouillard et al., 2016). These findings, which were included in the original manuscript, strongly support the view that Mef2 is a direct regulator of pum transactivation in both insects and mammals. In mammals, protein-protein interaction between p300 (through TZA2 domain) and Mef2A/Mef2C (through MASD/Mef2 domains) has also been reported (Sartorelli et al., 1997; De Luca et al., 2003; He et al., 2011). A comparison of these two domains in human and Drosophila show 85% (TZA2 domains) and 86~89% (MASD/Mef2 domains) amino acid identify. These observations (also stated in the original manuscript) support our yeast two-hybrid data showing interaction between p300 and Mef2. Thus, we feel that to further discuss this issue is of little value. We have, however, added the following sentence to the revision to address this concern: 

Line 482: ‘However, validation of the interaction of Mef2 with its putative binding sites will require confirmation by additional approaches (e.g. chromatin immunoprecipitation or EMSA).’ 


2. If p300 and Mef2 can be expressed in bacteria and then purified, the above-mentioned EMSA performed in the presence of increasing amount of p300 might validate the direct inhibitory effect of p300 on MEF2's DNA binding activity. Moreover, with this approach it might possible to assess whether the Mef2 isoform containing the exon 10 – the isoform that shows the most robust transactivation activity - binds DNA with a more affinity. 

Although bacterial expression is a common expression system employed for the production of recombinant proteins, we are not able to predict whether p300 or Mef2 expressed in a prokaryotic based system will be correctly processed. It would be an interesting project to produce recombinant Mef2 isoforms to determine the relationship between Mef2 DNA binding affinity and transactivation activity of the dpum promoter. However, we feel that this is beyond the scope of this current study. Thus, we do not address this concern in the revision. 


3. Page 18: please modify the “Supplementary data are available at NAR online.” 

We have changed this statemenmt to (Line 577): ‘Additional supporting information can be found online in the Supporting Information section at the end of the article’. 


Reviewer: 2 
- A trivial, but helpful bioinformatics control: in similar-sized promoter regions of a random selection of genes, how many dMef2 elements are seen on average, and what is the standard deviation? 

We now include the following statement (Lines 453) ‘Comparison with genes known to be transactivated by dMef2 (e.g., myoD, inflated, mir-1, tubulin60D and others) identifies, on average, 5 ± 1.6 (SD) Mef2 binding motifs (Lin and Baines, unpublished data).’ 


- Line 263 – In this section, it is somewhat of a leap to conclude that dPum levels are what is affected (although likely), since the assay is indirect. Since other post-translational ways of activating Pum are possible, it would be more correct to say that dPum activity is increased. It may be relevant to mention that it is the activity of endogenous Pum that is being affected. 

We have changed to ‘We conclude that increasing dmef2 is sufficient to increase dPum protein activity, in addition to transcript’ (Line 325). 

- Line 697 – “dMef2 binding elements are required for dpum promoter transactivation and increased dPum activity”. This is an overstatement, since the binding elements themselves were not tested by mutagenesis, and large sections of the promoter region were deleted instead. Whether dMef2 binding elements are required for increased dPum activity was not really tested at all. 

We have changed the title of Fig. 4 as ‘dMef2 is sufficient to modify dpum promoter transactivation and dPum protein activity’ (Line 871) 


- Neuronal activity-dependent changes in dmef2 transcript levels are measured, but protein levels and phosphorylation status (which is important in other model systems) are not, so it is a stretch to conclude that dMef2 levels are not impacted by synaptic excitation in the results section. The possibilities of post-transcriptional control and effects of phosphorylation should be mentioned upfront. 

To address this concern we add the following sentence ‘We are not, however, able to rule out activity-dependent post-transcriptional and/or post-translational modifications of dMef2 which may, in turn, influence expression or activity of dPum.’ (Line 337). 

- Connections to the human regulatory model, where Mef2 induces miR-134 expression, thus repressing Pum2, could be discussed in further detail. Is there a drosophila homolog of miR-134, is there experimental evidence of dMef2 – miR-134 regulation? Are there dMef2 sites in the miR-134 promoter? 

There is no Drosophila homolog of miR-134 found in the mirbase (http://www.mirbase.org). Using an online microRNA analysis tool (microRNA.org), we identified miRNAs, including miR-314, -315, -375, -927, -956 and -964, to potentially target the dpum 3’UTR, but not miR-134. In the same way, we identified miR-134 targets the 3’UTR of mammalian Pum2. These observations suggest the regulation of pum, through microRNA, may differ between mammals and insects. However, a discussion of this issue is, we believe, beyond the scope of this current study. 


We hope that you will find our revision acceptable for publication.

2^nd^ Editorial Decision	21/12/18

_____________________________________________________________________________________

Myocyte enhancer factor-2 and p300 interact to regulate expression of the homeostatic regulator Pumilio in Drosophila 

Dear Dr. Baines, 

Your revised manuscript was re-evaluated by external reviewers as well as by the Section Editor, Dr. C. Giovanni Galizia and ourselves.  We are pleased to inform you that we expect that it will be acceptable for publication in EJN following further minor revision. 

Both referees have evaluated your revised manuscript, and find - as we do -  that the manuscript is now ready for publication. Referee number 2 has one comment, i.e. the question, how many dMef2 elements exist in a random selection of genes that are unlikely to be targeted by dMef2? This is an interesting question, and you might consider doing a bioinformatics analysis and adding this information. If you consider this beyond the scope of the present paper then please add brief comments to the paper about the issue.  Also, please read the manuscript carefully for small language errors that might be present (e.g. on page 3, line 65: there is an "is" to many, in the sentence "The number of transcripts expressing PREs is underscores the importance of Pum." 

When revising the manuscript, please embolden or underline major changes to the text so they are easily identifiable and please don't leave 'track change' formatting marks in your paper.  At this stage, please provide text and a figure file for the Graphical Abstract.  When carrying out your revisions please refer to the checklist below and visit the EJN author guidelines at www.ejneuroscience.org 

When finalised, please upload your complete revised manuscript onto the website as a Word (.doc or .docx), or .rtf file.  Please also ensure that a complete set of tables and figures is included as separate files, even if these have not changed from the originals.  At this stage it is necessary to provide high resolution figures.  Please see important instructions below. 

Please go into https://mc.manuscriptcentral.com/ejn - Author Centre - manuscripts with decisions where you will find a 'create a revision' link under 'actions'. We ask that you please indicate the way in which you have responded to the points raised by the Editors and Reviewers in a letter. Please upload this response letter as a separate Word (.doc) file using the file designation "Authors' Response to Reviewers" when uploading your manuscript files. Please DO NOT submit your revised manuscript as a new one.  Also, please note that only the  Author who submitted the original version of the manuscript should submit a revised version. 

If you are able to respond fully to the points raised, we shall be pleased to receive a revision of your paper within 30 days. 

Thank you for submitting your work to EJN. 

Best wishes, 

Paul Bolam & John Foxe 
co-Editors in Chief, EJN 

Reviews: 

Reviewer: 1 

Comments to the Author 
Although the revised version does not contain additional data, the authors have properly answered the comments of the reviewers. I think this manuscript is now acceptable for publication in EJN. 


Reviewer: 2 

Comments to the Author 
The authors have addressed my comments sufficiently, except the one below: 

What I meant by Comment 1 is the number of dMef2 elements in a random selection of genes that are unlikely to be targeted by dMef2: the background, negative control number of dMef2 elements to be expected. I believe this is still a relevant number to include. The information the authors provided - positive control numbers on known dMef2 targets - is useful as well. 

------------------------

_____________________________________________________________________________________

Authors’ Response

_____________________________________________________________________________________

Dear Editors 

We are pleased that our manuscript has been deemed suitable for publication. 

We have made the final change requested: 

Line 453….’ Comparison with genes known to be transactivated by dMef2 (e.g., myoD, inflated, mir-1, tubulin60D and others) identifies, on average, 5 ± 1.6 Mef2 binding motifs (analysis of 8 genes ± SD). By comparison, analysis of a similar number of genes with no reported regulation by Mef2 (e.g., tailup, cry, lim3, eve, shaker and 3 others) identifies 3.2 ± 1.4 binding motifs per gene (P = 0.009, unpaired t-test, Lin and Baines, unpublished data).’
